# Current barriers and recommendations on the diagnosis of transthyretin amyloid cardiomyopathy: a Delphi study

**DOI:** 10.3389/fcvm.2024.1299261

**Published:** 2024-01-25

**Authors:** Yüksel Çavuşoğlu, İbrahim Başarıcı, Omaç Tüfekçioğlu, Ebru Özpelit, Elif Özdemir, İlknur Ak Sivrikoz, Hakan Altay, Muzaffer Değertekin, İrem Dinçer, Barış İkitimur, Gökhan Kahveci, Murat Fani Bozkurt, Metin Erkılıç, Gamze Çapa Kaya, Meral Beksaç, Ayşe Salihoğlu, Lale Tokgözoğlu

**Affiliations:** ^1^ Department of Cardiology, Eskisehir Osmangazi University Medical Faculty Hospital, Eskisehir, Turkey; ^2^ Department of Cardiology, Akdeniz University Medical Faculty Hospital, Antalya, Turkey; ^3^ Department of Cardiology, University of Health Sciences Ankara City Hospital, Ankara, Turkey; ^4^ Department of Cardiology, Dokuz Eylul University Medical Faculty Hospital, Izmir, Turkey; ^5^ Department of Nuclear Medicine, Ankara Yildirim Beyazit University, Ankara City Hospital, Ankara, Turkey; ^6^ Department of Nuclear Medicine, Eskişehir Osmangazi University Medical Faculty Hospital, Eskisehir, Turkey; ^7^ Department of Cardiology, Baskent University Medical Faculty Hospital, Istanbul, Turkey; ^8^ Department of Cardiology, Yeditepe University Medical Faculty Hospital, Istanbul, Turkey; ^9^ Department of Cardiology, Ankara University Medical Faculty Hospital, Ankara, Turkey; ^10^ Department of Cardiology, Istanbul University Medical Faculty Hospital, Istanbul, Turkey; ^11^ Department of Cardiology, Başakşehir Çam Sakura City Hospital, Istanbul, Turkey; ^12^ Department of Nuclear Medicine, Hacettepe University Medical Faculty Hospital, Ankara, Turkey; ^13^ Department of Nuclear Medicine, Akdeniz University Medical Faculty Hospital, Antalya, Turkey; ^14^ Department of Nuclear Medicine, Dokuz Eylul University Medical Faculty Hospital, Izmir, Turkey; ^15^ Department of Internal Diseases, Division of Hematology, Ankara University Medical Faculty Hospital, Ankara, Turkey; ^16^ Department of Internal Diseases, Division of Hematology, Istanbul University Medical Faculty Hospital, Istanbul, Turkey; ^17^ Department of Cardiology, Hacettepe University Medical Faculty Hospital, Ankara, Turkey

**Keywords:** amyloidosis, transthyretin amyloid, cardiomyopathy, diagnosis, cardiac scintigraphy

## Abstract

**Objectives:**

This study has been conducted to investigate the non-invasive diagnostic journey of patients with a transthyretin amyloid cardiomyopathy (aTTR-CM) in Turkey, identify the challenges and uncertainties encountered on the path to diagnosis from the perspectives of expert physicians, and develop recommendations that can be applied in such cases.

**Methods:**

This study employed a three-round modified Delphi method and included 10 cardiologists and five nuclear medicine specialists. Two hematologists also shared their expert opinions on the survey results related to hematological tests during a final face-to-face discussion. A consensus was reached when 80% or more of the panel members marked the “agree/strongly agree” or “disagree/strongly disagree” option.

**Results:**

The panelists unanimously agreed that the aTTR-CM diagnosis could be established through scintigraphy (using either 99mTc-PYP, 99mTc-DPD, or 99mTc-HMPD) in a patient with suspected cardiac amyloidosis (CA) without a further investigation if AL amyloidosis is ruled out (by sFLC, SPIE and UPIE). In addition, scintigraphy imaging performed by SPECT or SPECT-CT should reveal a myocardial uptake of Grade ≥2 with a heart-to-contralateral (H/CL) ratio of ≥1.5. The cardiology panelists recommended using cardiovascular magnetic resonance (CMR) and a detailed echocardiographic scoring as a last resort before considering an endomyocardial biopsy in patients with suspected CA whose scintigraphy results were discordant/inconclusive or negative but still carried a high clinical suspicion of aTTR-CM.

**Conclusion:**

The diagnostic approach for aTTR-CM should be customized based on the availability of diagnostic tools/methods in each expert clinic to achieve a timely and definitive diagnosis.

## 1 Introduction

Cardiac amyloidosis (CA) was previously considered a rare form of restrictive cardiomyopathy leading to mortality, but it is now increasingly recognized as a contributing factor to heart failure (HF) in elderly patients, particularly those with preserved ejection fraction (1, 2). Light chain amyloidosis (AL) and transthyretin amyloid cardiomyopathy (aTTR-CM) are the most common forms encountered in CA diagnosis (3). aTTR-CM is further classified into two subtypes based on the sequence of the TTR protein: wild-type (wtTTR) and hereditary (vTTR), the latter resulting from genetic variants in the *TTR* gene. Although AL-CA is a rare condition with an estimated incidence of 8–12 cases per million individuals, emerging data suggest that aTTR-CM is not uncommon, particularly due to wtTTR (1, 4–6). Recent reports using contemporary diagnostic strategies estimate that the prevalence of wtTTR is as high as 10%–16% among older patients diagnosed with heart failure or aortic stenosis (7–9). However, CA in general remains an under detected cause of heart failure, which is associated with high mortality if not treated appropriately during the early stages of the disease (10).

In Turkey, the prevalence, incidence, and survival rates of heart failure were on par with those in Western countries. However, a notable difference was observed in the age of onset, with HF manifesting 8–10 years earlier in the Turkish population (11). Although there is currently a lack of epidemiological data on *TTR* mutations in Turkey, a recent diagnostic study using multicenter next-generation sequencing (NGS) technology was conducted at 23 centers across Turkey suggested that TTR mutations were rare in Turkey, with only one *TTR* mutation identified among 392 patients with hypertrophic cardiomyopathy (HCM) (12). Another multicenter, national, observational study examined 886 patients who applied to the cardiology clinics in 22 centers managed to identify 15 (1.69%) patients with CA who were diagnosed by endomyocardial biopsy (13). A prospective, observational, single-center study from Turkey also reported 15 patients (17.6%) with a positive specific scintigraphy result, confirming the presence of ATTR-CA among 85 patients with heart failure with preserved ejection fraction (HFpEF) (14).

Endomyocardial biopsy also has been described as the gold standard for the diagnosis of aTTR-CM by ESC 2021 guideline, with approximately 100% sensitivity and specificity only if specimens are collected from >4 multiple sites and tested for amyloid deposits using Congo red staining (15, 16). However, one biopsy result cannot eliminate amyloidosis possibility when there is a high clinical suspicion, and there can be false negative results from superficial and inadequately prepared and dyed samples. In addition, there are several concerns on serious acute or delayed complication risks of endomyocardial biopsies for patients such as perforation with pericardial tamponade, pneumothorax, and puncture of the central arteries (17). Several studies have attempted to assess the sensitivity and specificity of non-invasive methods for the diagnosis of aTTR-CM. Current literature indicates that establishing the diagnosis of aTTR-CM is possible primarily through the use of technetium-labeled cardiac scintigraphy [99mTc-pyrophosphate (99mTc-PYP), 99mTc-3,3-diphosphono-1,2-propanodicarboxylic acid (99mTc-DPD), and 99mTc-hydroxymethylene diphosphonate (99mTc- HMDP) scintigraphy]. This method involves planar and SPECT imaging and has a specificity and positive predictive value of up to 100% when the results of serum free light chain assay (sFLC) and serum and urine protein electrophoresis with immunofixation (SPIE and UPIE) excluded AL-CA (18).

The task force recommendations on the diagnosis and treatment of CA and guidelines for the diagnosis and treatment of acute and chronic heart failure published by the European Society of Cardiology (ESC) outlined and proposed similar diagnostic algorithms for aTTR-CM (16, 19). In addition, the multidisciplinary consensus published by the American Society of Nuclear Cardiology (ASNC) with expert representatives from the American College of Cardiology (ACC), the American Heart Association (AHA), the American Society of Echocardiography (ASE), the European Association of Nuclear Medicine (EANM), the Heart Failure Society of America (HFSA), the International Society of Amyloidosis (ISA), the Society for Cardiovascular Magnetic Resonance (SCMR), and the Society of Nuclear Medicine and Molecular Imaging (SNMMI) broadly defined the appropriate use and interpretation of echocardiography, cardiovascular magnetic resonance (CMR), and technetium-labeled cardiac scintigraphy in patients with an established or suspected CA diagnosis (20). Current literature and guidance on CA diagnosis also emphasize the importance of clinical context and the crucial need to exclude AL amyloidosis. However, the definitions and recommendations in the related guidelines and consensus studies were developed based on the assumption that all diagnostic methods are available in clinics; all quality, standardization, and accreditation requirements are met; and all specialists have ideal experience with these diagnostic criteria and imaging methods.

Despite that the recommendations from international expert societies provide important guidance to physicians for the diagnosis and treatment of diseases, they may fall short in offering solutions for situations where physicians have difficulty in establishing a diagnosis in actual clinical practice within the scope of capabilities of their clinics. The heterogeneity of patients with CA as well as the incapacity of diagnostic tools make it difficult to diagnose aTTR-CM.

These challenges lead to differences in diagnostic approach across different centers and significant delays to achieve accurate diagnosis in Turkey as well as in many other developed and developing countries (21). This study has been conducted to investigate the non-invasive diagnostic journey of patients diagnosed with aTTR-CM in Turkey, identify the challenges and uncertainties encountered on the path to diagnosis from the perspectives of expert physicians, and develop practical recommendations that can be applied in such cases.

## 2 Methods

This modified Delphi study was consisted of three rounds and conducted over a 3-month period (between 6 August 2021 and 28 October 2021). The Delphi method systematically and interactively brings together the opinions of independent experts in two or more rounds when there is a lack of sufficient data and limited information or recommendations on a given subject and may allow experts to make a joint decision or reach a consensus (22, 23). The number of rounds or participants the Delphi panel should involve has not been specified in any guideline since it may vary depending on the aim of the relevant study (24).

The classic Delphi technique, known for the repeated question and answer rounds, can be modified by combining this method with different activities (24). The modification elements applied with the aim of reaching a consensus in the present Delphi study included semi-structured interviews, literature search, repeated online surveys, and face-to-face discussion of results, respectively.

### 2.1 Development of questions and evaluation of answers

During the first stage of this Delphi panel (semi-structured interviews), the panel members were asked open-ended questions regarding their general experience on aTTR-CM and the current barriers to reach an aTTR-CM diagnosis in their own clinics. The physicians conveyed their comments without a time limit. Based on the interview outcomes, a systematic literature search was conducted to prepare the first-round questions by the same external expert consultant who conducted the semi-structured interviews, in order to avoid the response bias of the physicians participating in the study. The available literature published between 2010 and 2021 was searched online in MEDLINE (via the PubMed interface), Web of Science, Google Scholar and EMBASE databases by using “MESH (Medical Subject Heading, Medline)” and “EMBASE” terms as well as free text words. The search terms included “amyloidosis,” “cardiac” “transthyretin,” “TTR,” “light chain,” “AL,” “diagnosis,” “echocardiography,” “cardiac magnetic resonance imaging,” “scintigraphy,” “discordance,” “false negative,” “false positive,” and “non-invasive diagnosis.” As additional selection criteria, evidence-based recommendations and care pathways were highlighted. Relevant guidelines from 2010 to 2021 were systematically reviewed. The main topics to be investigated in the survey were then pooled, and questions were developed.

A total of 84 questions were asked to cardiologists and 36 questions were asked to nuclear medicine specialists during the two-stage online survey (Supplementary Appendix 1). Questions were prepared in the form of open- and closed-ended styles to explore the experience and observations of the panelists in patients whom they diagnosed with CA in the last 5 years, their personal approach to confirm an aTTR-CM diagnosis in clinical practice, and their perspective on the available literature including the latest ESC recommendations. A 5-point Likert scale was used to seek consensus on their statements based on literature and experience. It was considered a consensus when 80% or more of the panel members marked the “agree/strongly agree” or “disagree/strongly disagree” option. The questions asked in the second round were either the repetition of the first-round questions or new questions that were developed based on the additional information and feedback provided by the panelists during the first round (first- and second-round questions are provided as Supplementary Material). An observation of disagreement between the cardiologist and nuclear medicine specialist groups for the joint questions was considered a reason for non-consensus.

### 2.2 Participants and the Delphi process

A total of 10 cardiologists who were experienced in cardiac amyloidosis and involved in the ongoing Prevalence and Prognosis of Cardiac Amyloidosis in Turkey (PAPCAT) registry study and five nuclear medicine specialists who had significant imaging experience in this field and participated in the aforementioned registry were chosen as active panel members in the current Delphi study (20). All panelists were either from a university or a research and training hospital. The distribution of involved centers across Turkey were as follows: Istanbul (4), Ankara (3), Antalya (1), Eskisehir (1) and Izmir (1), which was found to be a fair presentation based on patient population distribution among big cities in Turkey. These 15 experts shared their opinions and experience through semi-structured interviews and answered online survey questions in two rounds. The nuclear medicine specialists were only interviewed and surveyed on the scintigraphy-related topics.

Barriers to diagnostic tools and methods in clinical practice were identified during the semi-structured interviews and from the results of the first-round online survey. Practical recommendations to overcome these barriers and reach an accurate diagnosis in CA were then developed by considering the available clinical resources in Turkey, and a consensus was sought in accordance with the Delphi method during the second round of the online survey. After the evaluation of the online survey results, all panel members gathered in a face-to-face meeting. During the face-to-face meeting, two hematologists who were experienced in the field of amyloidosis shared their expert opinions on the survey results related to hematological tests. Finally, all panel members expressed their final opinions on the outputs.

## 3 Results

The online survey results and notes from the face-to-face discussion with 50% or higher agreement percentage are presented in this section under three headings: diagnostic tool/method preferences of cardiologists in patients with suspected CA, current barriers and practical recommendations to reach an accurate aTTR-CM diagnosis by cardiac scintigraphy, and the tools/methods to be used in patients with discordant or equivocal scintigraphy results.

### 3.1 Diagnostic tool/method preferences of cardiologists in patients with suspected CA

During the half-structured interviews and final face-to-face discussions, majority of the cardiologists stated that they usually refer to scintigraphy and hematological tests concurrently as soon as they have a clinical suspicion of CA. Few cardiologists who work with experienced radiologists in CA also stated that this concurrent referral includes a CMR imaging as well. However, when they had to put their diagnostic tool/method preferences in an order to assess a patient with suspected CA (based on clinical, ECG, and ECO findings), 70% of the cardiologist panel members chose the hematological tests first to investigate AL and chose the bone scintigraphy as the most second preferred method (50%, *n*: 10) (Table 1).

**Table 1:** Diagnostic tool preference order of the cardiologist panelists^a^.

Diagnostic tool	Preference order						
	1	2	3	4	5	6	I do not use this method
Hematological tests (serum and urine) for differential diagnosis of AL amyloidosis	70.00%	20.00%	0.00%	0.00%	10.00%	0.00%	0.00%
	7	2	0	0	1	0	0
Bone scintigraphy	20.00%	50.00%	30.00%	0.00%	0.00%	0.00%	0.00%
	2	5	3	0	0	0	0
Cardiac biopsy	0.00%	0.00%	0.00%	20.00%	10.00%	40.00%	30.00%
	0	0	0	2	1	4	3
Extracardiac biopsy	0.00%	0.00%	10.00%	40.00%	30.00%	10.00%	10.00%
	0	0	1	4	3	1	1
Cardiac MRI (CMR)	10.00%	30.00%	30.00%	10.00%	10.00%	10.00%	0.00%
	1	3	3	1	1	1	0
Genetic testing	0.00%	0.00%	30.00%	30.00%	20.00%	10.00%	10.00%
	0	0	3	3	2	1	1

^a^ Related panel question: Which of the following diagnostic tools and in which order do you usually refer to while investigating an aTTR-CM presence in a patient with a suspected CA based on ECG and echocardiography findings? (Please list your diagnostic tool preference according to your own clinic's conditions and capabilities. You may exclude the methods you do not use by clicking the box on the right for each option).

All cardiologist panel members stated that if sFLC, SPIE, and UPIE results excluded AL, they would then refer to bone scintigraphy as the next diagnostic step. While cardiologists fully agreed on the diagnostic flow provided in the ESC's recent position statement paper, they also agreed on the new additional statements underlining the immediate need to refer patients with specific findings (red flags) to advanced clinics (if the unit is lacking infrastructure) and the need for multidisciplinary collaboration between cardiology, hematology, and nuclear medicine.

**Figure 1:**
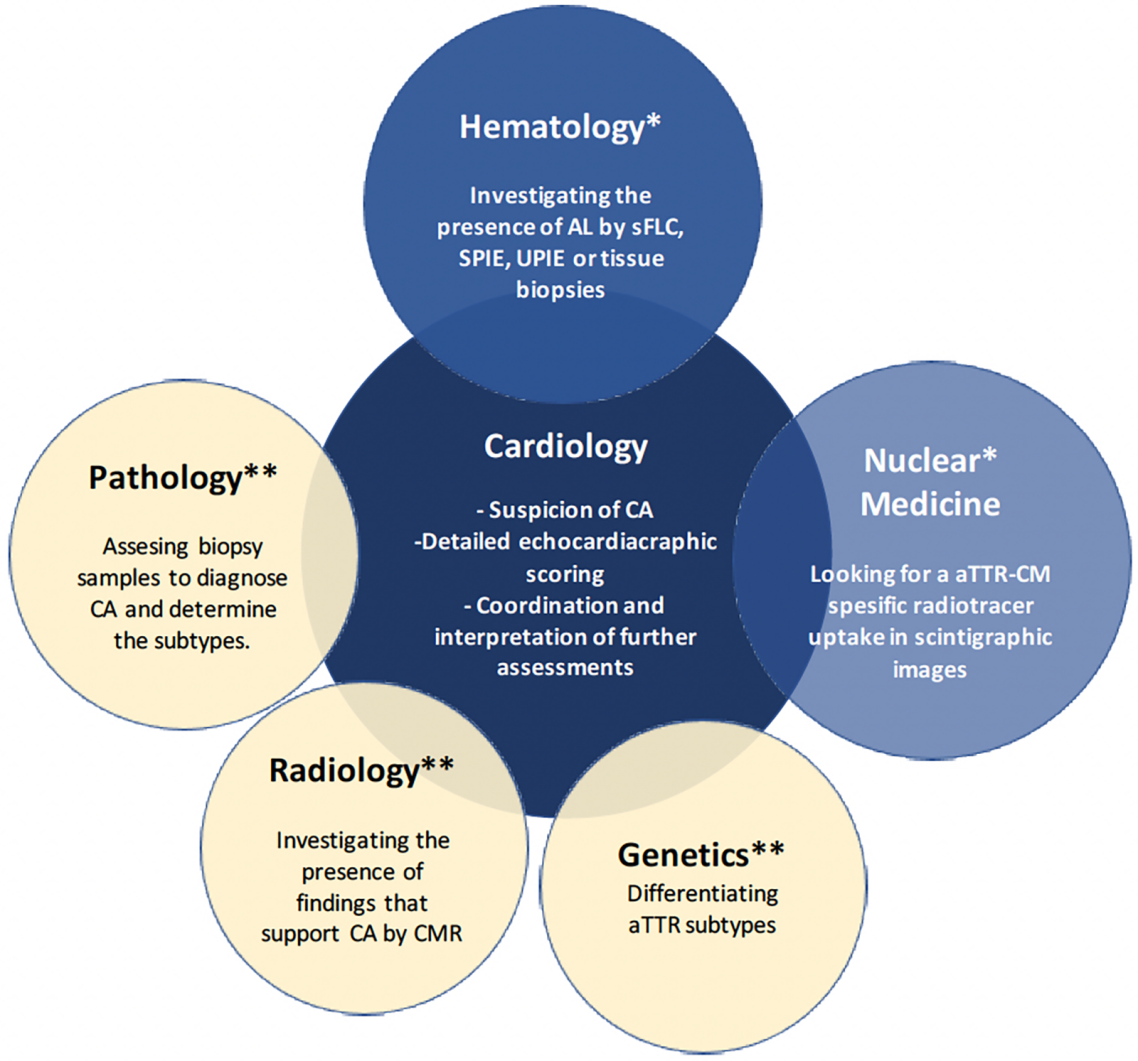
Importance of the multidisciplinary approach in aTTR-CM diagnosis: *essential referral units for the non­invasive diagnosis of aTTR-CM, **required referral units when the diagnosis of aTTR-CM is not possible by non-invasive methods.

While the diagnostic tool/method preference orders of the cardiologist panelists were as mentioned in Table 1 and Figure 1, the methods they used within the last 5 years in their clinical practice to confirm the aTTR-CM diagnosis at the last step were either scintigraphic imaging with 99mTc-PYP, CMR, or extracardiac biopsies with a similar preference frequency. In addition, 50% of the cardiologist panelists stated that they requested genetic testing for each aTTR-CM patient they diagnosed, and 20% of them reported that they did not refer to genetic testing in any of their diagnosed patients.

### 3.2 Current barriers and practical recommendations to exclude AL and to reach an accurate aTTR-CM diagnosis by cardiac scintigraphy

#### 3.2.1 Serum free light chain assay and serum and urine protein electrophoresis with immunofixation in ruling out AL amyloidosis

While majority of the cardiologist panel members agreed on the access difficulties to sFLC, SPIE, and UPIE to exclude AL amyloidosis and lack/absence of experts in their clinics to interpret test results, a consensus could not be reached to label this statement as a barrier for clinics in Turkey. During the face-to-face discussions, guest hematologist experts shared the panelists' concerns on the access difficulties to sFLC, SPIE, and UPIE in clinics. They also underlined the absolute need for a hematologist opinion particularly to differentiate a monoclonal gammopathy of undetermined significance (MGUS) from other malignant plasma cell disorders such as multiple myeloma when a monoclonal gammopathy is observed. Despite the mentioned setbacks regarding the periodic kit supply or specialist availability for the accurate interpretation of sFLC, SPIE, and UPIE test results, the cardiology panel members agreed that these barriers could be overcome by collaborating with external clinics or private laboratories.

The following recommendation statements were then developed and achieved consensus: sFLC, SPIE, and UPIE can be requested in patients with suspected CA without resorting to CMR imaging; sFLC, SPIE, and UPIE should be concluded before or concurrently with bone scintigraphy if these AL differentiating tests are accessible and can be finalized within a reasonable time; and if not, bone scintigraphy should be prioritized and performed without a delay. Panelists fully agreed and underlined that a definitive diagnosis should not be established in any case until the hematological tests used for the differential diagnosis of AL are completed (Table 2).

**Table 2:** Consensus statements for AL diagnostic tests in clinical practice.

Barriers to exclude AL amyloidosis	Cardiology consensus rate %^a^
Periodic or permanent access difficulties to sFLC, SPIE, and UPIE, periodic test kit supply problems, and lack of specialists in clinics to interpret test results form a barrier to exclude AL amyloidosis.	60
Recommendations to obtain a timely and accurate AL amyloidosis assessment	Cardiology Consensus rate %^a^
Clinical, ECG, and echocardiography findings of the patients are often satisfactory to suspect CA; therefore, patients with suspected CA can directly be referred to sFLC, SPIE, and UPIE without resorting to CMR.	100
In a patient with suspected CA (based on clinical, ECG, and echocardiography findings), sFLC, SPIE, and UPIE tests should be concluded before or concurrently with bone scintigraphy if it is possible to access these tests and their interpreter specialists within a reasonable time.	90
Bone scintigraphy should be performed without a delay in patients with suspected CA (based on clinical, ECG, and echocardiography findings) if sFLC, SPIE, and UPIE results excluded AL, or when delayed access/results are likely for sFLC, SPIE, and UPIE.	90
The absence of a monoclonal gammopathy by sFLC, SPIE, and UPIE mostly exclude AL amyloidosis.	100
A definitive diagnosis should not be established in patients with suspected CA until sFLC, SPIE, and UPIE are concluded, regardless of bone scintigraphy result.	90
Difficulties to access AL tests or interpret test results could be overcome by collaborating with external clinics or private laboratories.	100

^a^ Consensus was defined as when 80%–100% of the panel members marked the “agree/strongly agree” or “disagree/strongly disagree” option.

#### 3.2.2 Technetium-labeled cardiac scintigraphy

While the cardiology panelists agreed that scintigraphy can also be referred to in patients with suspected CA without resorting to CMR imaging, cardiology and nuclear medicine specialist panelists also agreed on the statements concerning the barriers to access suitable radiotracers and accurate scintigraphy imaging in their clinics to diagnose aTTR-CM (90%, *n*: 10, 80% *n*: 5). During the half-structured interviews, the nuclear medicine specialists underlined that the current scintigraphy reimbursement scheme statement does not involve the use of specific suitable radiotracers to diagnose aTTR-CM and the lack of this point in the statement may interrupt the procurement of the specific radio tracers for the clinics. Therefore, all panelists reached a full consensus on the recommendation stating that “Revision of the current scintigraphy reimbursement scheme statement by including the use of specific radiotracers in patients with suspected aTTR-CM, will facilitate the supply of these radiotracers.”

In addition, full consensus was reached on the recommendations regarding the technical spadework that should be performed prior to conducting scintigraphy imaging (Table 3). All panelists agreed on the statement that all three radiotracers (99mTc-PYP, 99mTc-DPD, 99mTc-HMPD) can be used in scintigraphy imaging for the diagnosis of aTTR-CM. The panelists also underlined that experts should carefully consider the timing of imaging that is specific to each agent. Other statements in the technical details section with full consensus were as follows: single-photon emission computed tomography (SPECT) imaging should be performed after planar imaging to confirm the presence of myocardial uptake and prevent misinterpretations in aTTR-CM diagnosis, the ideal scintigraphy approach to investigate aTTR-CM is imaging at the first and third hour by using SPECT imaging with computed tomography (SPECT-CT), and blood pool activity (uptake) should be ruled out while evaluating these images (Table 3).

**Table 3:** Consensus statements for cardiac scintigraphy in clinical practice.

Barriers to accurate imaging in scintigraphy	Cardiology consensus rate %^a^	Nuclear medicine consensus rate %^a^
There are limitations in suitable radiotracer access for the diagnosis of aTTR-CM in secondary and tertiary healthcare facilities that are capable of performing scintigraphy in Turkey.	90	80
Lack of SPECT-CT at nuclear medicine clinics in Turkey is a barrier in terms of reducing the possibility of misinterpretations of scintigraphy in aTTR-CM diagnosis.	90	80
Visual assessments of scintigraphy images to confirm an aTTR-CM diagnosis are influenced by the reporting specialist's experience.	90	80
Improperly bound radiotracers, absorbed radiotracer activity dose measured by uncalibrated tools, failing to pay attention to the expiry date of the radiotracer, and interpreting images without paying attention to the quality control parameters of devices may cause errors in aTTR-CM diagnosis.	100	100
Recommendations to achieve accurate imaging in scintigraphy		
Revision of the current scintigraphy reimbursement scheme statement by including the use of specific radiotracers in patients with suspected aTTR-CM will facilitate the supply of these radiotracers.	100	100
Proper calibration, optimization, and quality controls of radiotracers and imaging devices (gamma camera, SPECT, or SPECT-CT devices) should be performed regularly.	100	100
All three radiotracers (99mTc-PYP, 99mTc-DPD, 99mTc-HMPD) can be used in scintigraphy imaging for the diagnosis of aTTR-CM.	100	100
Experts should carefully consider the timing of the imaging that is specific to each agent.	100	100
SPECT imaging should be performed after planar imaging to confirm myocardial uptake and prevent misinterpretations in aTTR-CM diagnosis.	100	100
The ideal scintigraphy approach to detect aTTR-CM is imaging at the 1st and 3rd hour by using SPECT-CT.	80	100
Blood pool activity (uptake) should be ruled out while evaluating scintigraphic images	100	100

^a^ Consensus was defined as when 80%–100% of the panel members marked the “agree/strongly agree” or “disagree/strongly disagree” option.

The panelists achieved full consensus on the following statements: A diagnosis of aTTR-CM can be established by scintigraphy in a patient with suspected CA without a further investigation (based on clinical, ECG, and ECO findings) when AL amyloidosis is ruled out (by sFLC, SPIE, and UPIE) and the scintigraphy result showed Grade ≥2 myocardial uptake with a heart-to-contralateral (H/CL) ratio of ≥1.5 confirmed by SPECT or SPECT-CT. There was a substantial increase in both disciplines' consensus strengths regarding their SPECT-CT preference (25%) (Table 4).

**Table 4:** Consensus statements for aTTR-CM diagnosis by scintigraphy.

The role of scintigraphy in aTTR-CM diagnosis	Cardiology consensus rate %^a^	Nuclear medicine consensus rate %^a^
Clinical, ECG, and echocardiography findings of the patients are often satisfactory to suspect CA; therefore, patients with suspected CA can directly be referred to scintigraphy without resorting to CMR.	100	NA
If there is access to experienced radiology specialists and CMR for CA evaluation, patients with suspected CA^b^ should be referred to CMR before or concurrently with scintigraphy.	70	NA
A diagnosis of aTTR-CM can be established* by scintigraphy in a patient with suspected CA^b^ without a further investigation, in whom AL amyloidosis has been ruled out^c^ and showed Grade ≥2 myocardial uptake with an H/CL ratio of ≥1.5 confirmed by SPECT**.	100	100
A diagnosis of aTTR-CM can be established* by scintigraphy in a patient with suspected CA^b^ without a further investigation in whom AL amyloidosis has been ruled out^c^ and showed Grade ≥2 myocardial uptake with H/CL ratio of ≥1.5 confirmed by SPECT-CT**.	100^d^	100^d^
Scintigraphy should be repeated by using SPECT-CT (if there is access within or outside the clinic), in a patient with suspected CA^b^, in whom AL amyloidosis has been ruled out^c^ and whose cardiac scintigraphy showed inconsistent findings on radiotracer uptake for aTTR-CM (Grade ≥ 2 with H/CL <1.5, or Grade <2) assessed only by SPECT.	90	80
Scintigraphy should be repeated by using 99mTc-PYP before ruling out the aTTR-CM diagnosis in a patient with suspected CA, in whom AL amyloidosis has been ruled out^c^ and whose cardiac scintigraphy performed by another radiotracer showed an inconsistent radiotracer uptake for aTTR-CM (Grade ≥2 with H/CL <1.5, or Grade <2).	50^e^	60^e^

* Wording difference in consensus statements of nuclear medicine specialist as aTTR-CM differentiation can be achieved in a patient with suspected CA^b^, in whom AL amyloidosis has been ruled out^c^ and showed Grade ≥2 myocardial uptake with an H/CL ratio of ≥1.5 confirmed by SPECT or SPECT-CT.

** In an optimized/calibrated technical setting.

^a^ Consensus was defined as when 80% to 100% of the panel members marked the “agree/strongly agree” or “disagree/strongly disagree” option.

^b^ Based on clinical, ECG, and ECO findings.

^c^ By sFLC, SPIE, and UPIE.

^d^ The strength of the consensus was increased by 25% for the SPECT-CT compared with SPECT.

^e^ %50 of cardiologists agreed and 60% of nuclear medicine specialists disagreed with the statement.

During the face-to-face discussions of this section, the panelists also shared the most encountered characteristics of their previous patients with suspected CA that led to false positive results with scintigraphy. These were the presence of concurrent or sole AL amyloidosis, rib fracture, valvular annular calcification, acute or subacute myocardial infarction, pericardial and/or pleural fluid, large breast structure, and non-homogeneous structure of the thoracic soft tissue.

### 3.3 The tools/methods to be used in patients with suspected CA who have discordant or inconclusive scintigraphy results for aTTR-CM, current barriers, and practical recommendations

#### 3.3.1 CMR and echocardiography

The cardiology panelists recommended using CMR and a detailed echocardiographic scoring as a last resort before considering an endomyocardial biopsy in patients with suspected CA whose scintigraphy results were discordant/inconclusive or negative but still carried a high clinical suspicion of aTTR-CM (Table 5). The reason for positioning CMR at this stage was stated to be the shortage of experienced radiologists in CA, which was claimed to constitute a significant barrier to confirm CA with CMR in the clinics. Similarly, the reason to position a detailed echocardiographic scoring at this stage was explained by the insufficient number of cardiologists even for the basic echocardiographic assessments.

**Table 5:** Consensus statements on CMR and detailed echocardiographic scoring.

Barriers to detect and differentiate CA with CMR in clinical practice	Cardiology consensus rate %^a^
Lack of experienced radiologists in CA constitutes a challenge to detect amyloidosis by CMR in clinical practice.	100
CA diagnosis can strongly be supported by CMR, yet it doesn’t have a defined ability to differentiate subgroups in amyloidosis.	100
Recommendations on when to use CMR, detailed echocardiographic scoring and biopsy in CA assessment	Cardiology consensus rate %*
A patient with a high clinical suspicion of CA^b^ should be evaluated by CMR^c^ before planning an endomyocardial biopsy, if he/she showed a discordant/inconclusive finding on radiotracer uptake for aTTR-CM or if scintigraphy results were negative (Grade ≥ 2 with an H/CL ratio of <1.5, or Grade 1 or Grade 0) but showed a high clinical suspicion during SPECT or SPECT-CT imaging.	100
A patient with suspected CA^b^ should be evaluated by detailed echocardiographic scoring before planning an endomyocardial biopsy, if he/she showed a discordant/inconclusive finding on radiotracer uptake for aTTR-CM or if scintigraphy results were negative (Grade ≥ 2 with H/CL <1.5, or Grade 1 or Grade 0) but showed a high clinical suspicion during SPECT or SPECT-CT imaging, unless performed previously.	100

^*^ If the patient's condition and evaluation capability of the unit are both suitable.

^a^ Consensus was defined as when 80% to 100% of the panel members marked the “agree/strongly agree” or “disagree/strongly disagree” option.

^b^ Based on clinical, ECG and ECO findings.

^c^ If there is access to experienced radiology specialists and CMR for CA evaluation.

#### 3.3.2 Endomyocardial or extracardiac biopsy

The cardiology panelists reached a consensus on that an endomyocardial or extracardiac biopsy can be performed in patients with suspected CA whose cardiac scintigraphy showed a discordant/inconclusive or negative result for aTTR-CM, by using SPECT or SPECT-CT, while clinical findings, CMR, and/or detailed echocardiographic scoring are still supporting the presence of CA. The reasons for positioning the endomyocardial biopsy as the last resort were claimed to be the current barriers to the accurate evaluation of biopsy samples by using immunohistochemical staining, both in terms of limited dye portfolios and evaluation sensitivity. The cardiologists fully agreed on the notion that there will be no need for a cardiac biopsy in clinics where non-invasive diagnostic tools for aTTR-CM are available. This consensus was established to avoid the potential risks/complications for the patient and due to the previously mentioned limitations in biopsy sample assessments (Table 6).

**Table 6:** Consensus statements on biopsy in clinical practice.

Barriers to accurate evaluation of samples obtained from biopsies	Cardiology consensus rate %*
There are technical deficiencies in defining the pathological characterization of CA biopsy samples in Turkey (regarding the sensitivity and limitations in the dye portfolio and unavailability of mass spectrometric analysis of biopsy specimens.)	100
Recommendations on when to use biopsy in aTTR-CM diagnosis	Cardiology Consensus Rate %*
An endomyocardial/extracardiac biopsy can be performed*, in a patient with suspected CA^a^, whose cardiac scintigraphy showed a discordant/inconclusive finding on radiotracer uptake for aTTR-CM or if scintigraphy results were negative (Grade ≥ 2 with H/CL <1.5, or Grade1 or Grade 0) assessed by SPECT or SPECT-CT, while CMR findings and/or detailed echocardiographic scoring is supporting the presence of a CA.	80
There is no need for a cardiac biopsy in clinics where non-invasive diagnostic tools for aTTR-CM are available due to the risks for the patient and the barriers in evaluation.	100
If biopsy is required to diagnose CA, extracardiac biopsies should be performed as the primary method.	100
For extracardiac biopsies, the sample may be obtained from abdominal fat tissue initially, then from the minor salivary gland or rectum if no results are obtained with the initial approach.	100

* If the patient's condition and evaluation capability of the unit are both suitable.

^a^ Based on clinical, ECG, and ECO findings.

In addition to the hematologic tests, technetium-labeled cardiac (bone) scintigraphy, CMR, detailed ECO scoring, and biopsy, the cardiology panelists also agreed on the limited access to genetic tests (including outsourcing) in healthcare facilities in Turkey. While panelists did not find genetic testing essential in the diagnostic journey of aTTR-CM patients, they suggested that the access conditions to genetic testing for CA should be improved to see the prevalence and diversity of mutations in the Turkish population and to prevent patients from being deprived of mutation-specific treatments in the future (consensus rate of 100%).

## 4 Discussion

**Figure 2:**
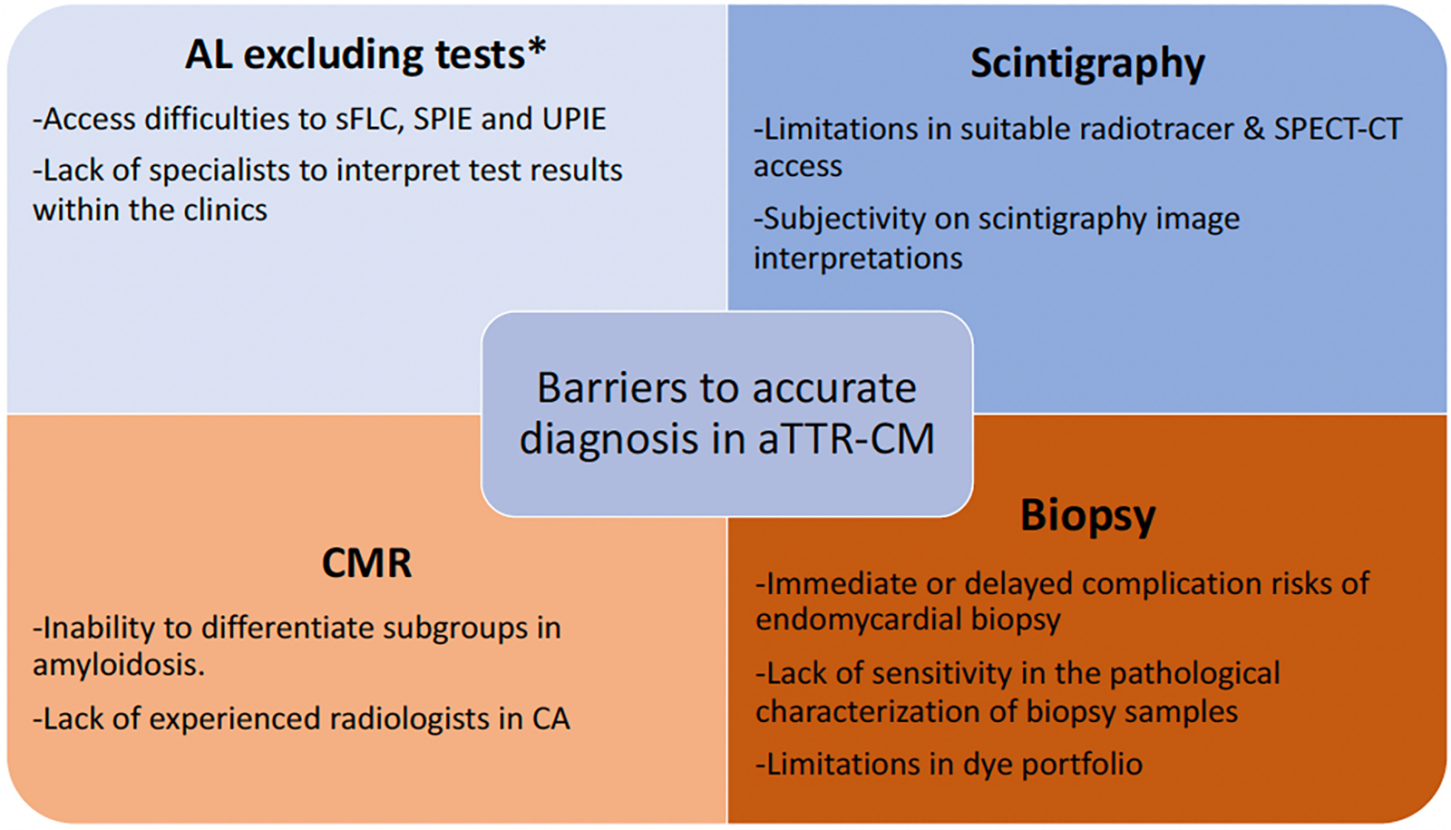
Summary of the main barriers to accurate diagnosis in aTTR-CM: *while the majority of the cardiologist panel members agreed on these barriers, a consensus could not be reached.

**Figure 3:**
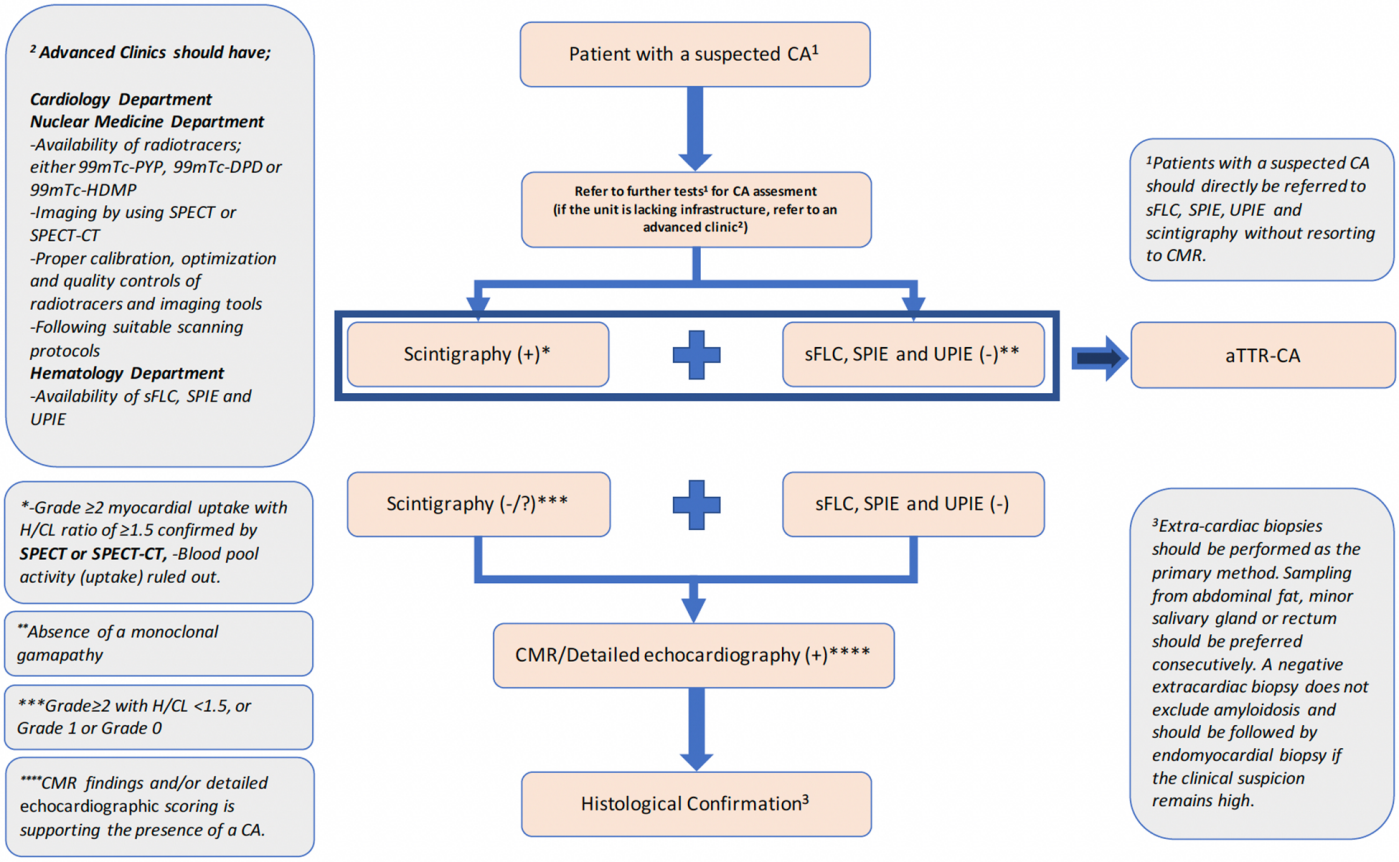
Diagnostic algorithm recommendation for aTTR-CM.

While there were no objections from the panelists to the most recent ESC and ASNC multidisciplinary consensus papers (19, 20), there were considerable differences among the diagnostic tool/method preference order of the cardiology panelists and previous diagnostic approaches in patients with suspected CA. The reasons behind these variations were found out to be different daily challenges in their clinical practice. In this study, the panelists also managed to achieve consensus on a substantial number of practical recommendations to overcome these challenges. During the establishment of these recommendation statements, the clinical availability of diagnostic tests/methods were put into consideration with a realistic perspective unlike in the guidelines, where each test/method was assumed to be easily accessible and each cardiologist/nuclear medicine/hematology/radiology specialist was considered to be experienced enough in CA and present in all clinics. The outputs of this panel are summarized in Figures 1–3 and approved by all panelists.

This study underlined the referral need of patients with CA suspicion (red flags) to advanced centers both in terms of their available diagnostic resources and specialists. A recent prospective, observational, single-center study from Turkey that aimed to evaluate the frequency of cardiac and extracardiac manifestations of CA in patients with heart failure with HFpEF based on red flags also confirms the necessity of timely expert center referrals as the study reported that patients diagnosed with HFpEF had an average of 1.3 red flags suggestive of CA (D-14). The same study also reported that patients with ATTR-CA had twice as many red flags as those without (2.46 vs. 1.04), and ATTR-CA diagnosis was more common in patients with 2 or more red flags (14).

The panelists reached a full consensus on the statement that recommends sFLC, SPIE, and UPIE tests to be concluded before or concurrently with bone scintigraphy if these tests are thought to be accessed within a reasonable time. However, they also agreed that bone scintigraphy should be performed first when delayed access/results are likely for hematological tests. Available evidence and guidance suggest that an accurate interpretation of scintigraphy in patients with suspected CA requires serum/urine testing to exclude a monoclonal immunoglobulin or light chain abnormality (18, 26, 27). Currently, the most efficient and effective approach to exclude these abnormalities is through three laboratory tests: IFE of the serum and urine and the sFLC assay (28, 29). If there is no monoclonal protein on IFE of the serum and urine and the sFLC assay presents with a normal ratio, then the negative predictive value for excluding AL amyloidosis claimed to be approximately 99% (28, 29). However, tissue biopsy with certain processing claimed to be necessary to establish the accurate diagnosis in many cases (30). These observations also underscore the importance of close collaboration with an experienced hematologist in AL amyloidosis.

All panelists agreed that aTTR-CM diagnosis could be established by scintigraphy (using either 99mTc-PYP, 99mTc-DPD, or 99mTc-HMPD) in a patient with suspected CA (based on clinical, ECG, and echocardiography findings) without a further investigation if AL amyloidosis is ruled out (by sFLC, SPIE, and UPIE) and scintigraphy imaging performed by SPECT or SPECT-CT showed Grade ≥2 myocardial uptake with an H/CL ratio of ≥1.5. These consensus statements are in line with the current guidelines/statement papers since they all propose a diagnostic algorithm focusing on identifying CA subtypes by the initial use of 99mTc-PYP, DPD, or HMDP scintigraphy coupled to assessment for monoclonal proteins by SPIE, UPIE, and quantification of sFLC (16, 19, 20). In addition, a Perugini score of Grade ≥2 and the absence of a monoclonal protein in serum and urine were previously shown to have a specificity of 100% for the diagnosis of aTTR-CM and a heart-to-contralateral ratio of ≥1.5 at 1 h on planar imaging demonstrated a high diagnostic accuracy for aTTR-CM, obviating the need for endomyocardial biopsy (18, 31, 32). It should be noted that the quantitative score is based on the H/CL ratio with 99mTc-PYP, which is a validated method (20). In this study, we did not assess nor discuss the score of the heart-to-whole body ratio (H/WB) for 99mTc-DPD and 99mTc-HDMP-based assessments as H/WB is not a validated scoring method nor directly recommended by guidelines.

While ASNC consensus recommendations suggests interpreting CT only for attenuation correction (16), the ideal scintigraphy approach to confirm an aTTR-CM diagnosis in this panel is defined as imaging at the first and third hour by using SPECT-CT imaging. In addition, ruling out blood pool activity (uptake) while evaluating scintigraphy images was claimed to be an essential part of the image interpretation. There is a theoretical possibility that focal radiotracer uptake may suggest that early aTTR-CM could be missed on SPECT, and blood pool activity could mask early/focal uptake; therefore, hybrid imaging with SPECT-CT could improve the diagnostic accuracy in such cases (33–36). In this panel, the limitations in suitable radiotracer and SPECT-CT access in secondary and tertiary healthcare facilities were reported as barriers to get the best performance out of scintigraphy as a diagnostic method for aTTR-CM in Turkey. Therefore, the panelists claimed that revision of the current scintigraphy reimbursement scheme statement by including the specific usage of certain radiotracers in patients with suspected aTTR-CM will facilitate the supply of suitable radiotracers.

When it comes to the positioning of CMR in CA assessment, the cardiology panelists agreed on the literature-based statement that underlines the detection ability of CMR in CA without its subgroup differentiation capacity (37–40). ESC and ASNC multidisciplinary consensus papers position CMR and detailed echocardiography scoring in aTTR-CM diagnosis with a notion of similarity by assuming that all imaging methods are available and all specialists have ideal experience in the clinics (16, 19, 20). However, the lack of experienced radiologists in CA constitutes a challenge to detect amyloidosis by CMR in clinical practice from the panelists' perspectives. Therefore, all cardiologists in this panel agreed that clinical, ECG, and echocardiography findings of the patients are often sufficient to suspect CA; therefore, patients with suspected CA can directly be referred to sFLC, SPIE, UPIE, and scintigraphy without resorting to CMR. However, the panelists stated that if a patient with suspected CA showed a discordant uptake for aTTR-CM or no radiotracer uptake (Grade ≥2 with H/CL <1.5, Grade 1, or Grade 0) during the scintigraphy imaging by SPECT or SPECT-CT, the patient should be then evaluated by CMR or detailed echocardiography scoring to confirm the presence of CA before planning an endomyocardial biopsy for histological confirmation.

The risks of endomyocardial biopsy were previously reported as acute or delayed. The immediate risks of biopsy include perforation with pericardial tamponade, ventricular or supraventricular arrhythmias, heart block, pneumothorax, puncture of central arteries, pulmonary embolization, nerve paresis, venous hematoma, damage to the tricuspid valve, and creation of arterial venous fistula within the heart (17, 41–43). Since most complications are known from case reports, the precise frequency of these events is not known. The risks of endomyocardial biopsy will mainly vary with the experience of the operator and the clinical status of the patient (17). In this panel, the cardiologist panelists agreed that if biopsy is required for the differential diagnosis of CA, extracardiac biopsies should be performed as the primary method; endomyocardial biopsy should be performed only if the evaluation yields equivocal or discordant findings for CA, and it should be avoided in patients who can be diagnosed by non-invasive diagnostic tools. In addition, as for extracardiac biopsies; abdominal fat, minor salivary gland, sural nerve, or rectum may be preferred consecutively, if no results are obtained at the initial approach. In cases where amyloidosis is suspected, there is evidence that by staining subcutaneous abdominal fat or rectal/labial salivary gland biopsies can confirm amyloidosis with a low risk and a 50%–80% sensitivity (44). Abdominal fat pad biopsies are more sensitive in detecting cardiac AL amyloidosis than aTTR. As for aTTR, while detection rates by abdominal fat pad biopsy samples vary from 45% to 67% for vTTR and 14% to 15% for wtTTR, skin (punch) biopsies were reported to provide better detection rates: 94% for vTTR and 63%–73% for wtTTR, as well as 91%–100% of vTTR detection rate with lip salivary gland samples (45). However, there is no data for the detection rates of wtTTR with minor salivary gland samples. Although the sensitivity of rectal biopsy to detect amyloidosis is reported approximately 69%–97%, to avoid false negative results (may reach 60%), rectal biopsies should include submucosal layer since amyloid deposits in patients with aTTR are settled mainly in the submucosa of the gastrointestinal tract (46). Only 16% of amyloid cases can be attributed to aTTR in patients with confirmed amyloidosis by gastrointestinal tract sampling (47). Also, when abdominal fat pad biopsy is negative, the diagnostic yield of rectal biopsy is so low that it is no longer suggested as an alternative site (45), while sural nerve biopsy gives promising results by detecting 80% of TTR amyloidosis particularly in patients with familial amyloid polyneuropathy (48). However, since amyloid deposits are not homogenously present on the entire length of the nerve, sural nerve biopsy is highly prone to sampling errors (46). Given the significantly differing and relatively low sensitivity of tissue biopsies, guidance papers state that a negative extracardiac biopsy does not exclude CA and should be followed by endomyocardial biopsy whenever the clinical suspicion remains high (44, 45, 49).

**Figure 4:**
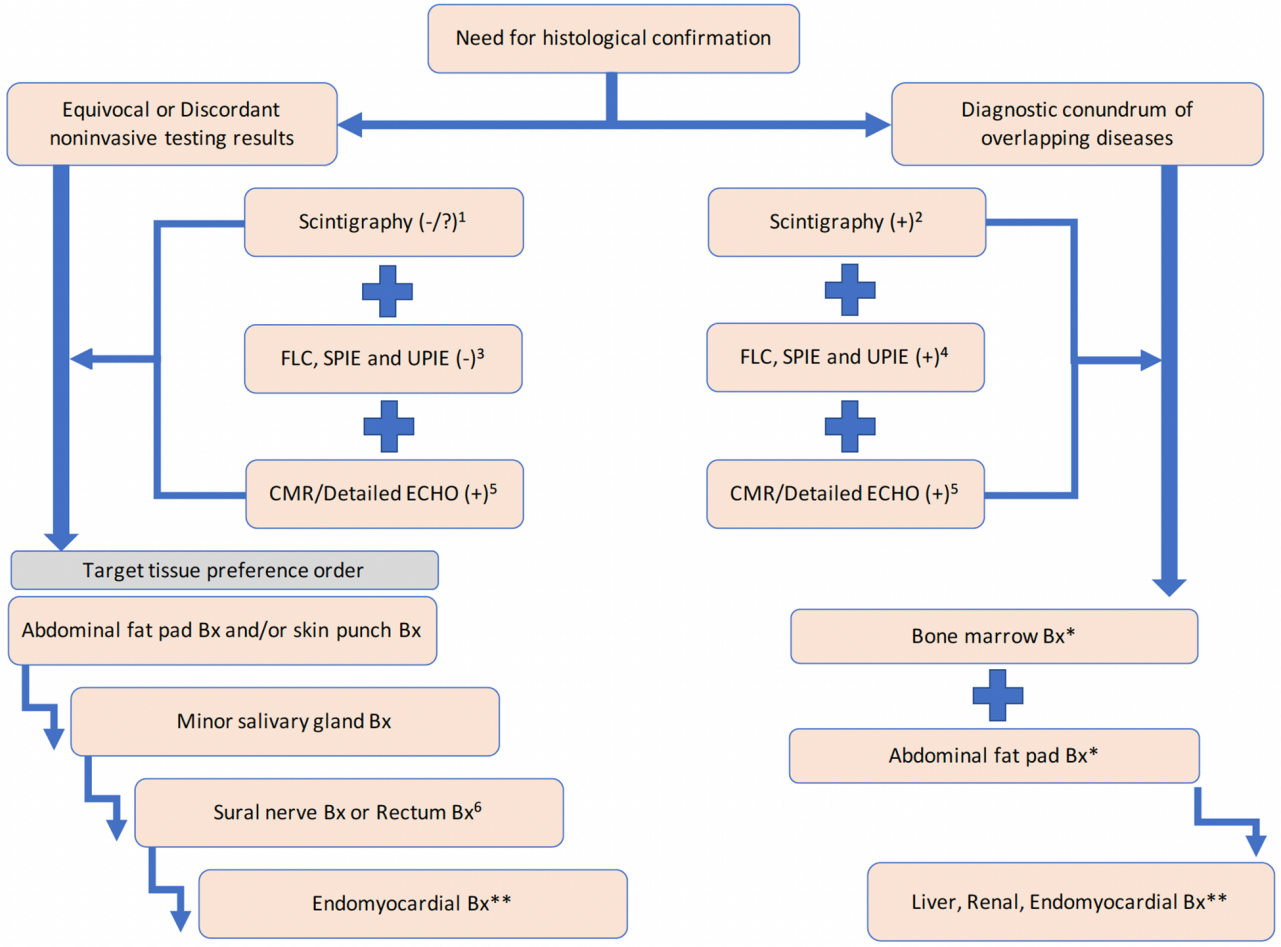
Histological confirmation algorithm recommendation when previous assessments came out as discordant or inconclusive for aTTR-CM: (1) Grade 2 with an H/CL ratio of <1.5, or Grade 1 or Grade 0; (2) Grade 2 myocardial uptake with an H/CL ratio of 1.5 confirmed by SPECT or SPECT-CT, blood pool activity (uptake) ruled out; (3) Absence of a monoclonal gammopathy; (4) Presence of a monoclonal gammopathy; (5) CMR finding and/or detailed ECHO scoring is supporting the presence of a CA; (6) Both have inherited drawbacks in the detection of ATTR and so this step can be skipped. *Bone marrow biopsy and abdominal fat pad biopsy should be performed first. **Biopsy of the clinically involved organs should be the last resort.

The panelists also notified that there may be rare but special subset of patients who are generally elderly and may have overlapping wtTTR and/or subclinical myeloproliferative disease presenting as smoldering myeloma or monoclonal gammopathy of undetermined significance. The detection of MGUS cannot warrant AL diagnosis, and up to 49% of patients with aTTR may have MGUS (50). For these instances of conundrum, confirmation of amyloidosis and histopathological characterization of effected tissues may be prioritized. So, two different algorithmic approaches can be offered for two different case scenarios requiring histological confirmation (Figure 4) according to the abovementioned needs and concerns. In the first case scenario, endomyocardial biopsy should be the last resort in the step-by-step approach where all other extracardiac biopsy results are negative in the setting of high clinical suspicion of CA. Biopsy of the iliac crest bone marrow combined with abdominal subcutaneous fat aspiration was previously shown to identify amyloid deposits in 85% of patients with amyloidosis (51). Therefore, on the other route, bone marrow biopsy and abdominal fat pad biopsy should be performed first to resolve the conflict aroused by the presence of monoclonal gammopathy, and biopsy of the clinically involved organs should be the last resort since renal and liver biopsy are expensive and invasive and there is an increased incidence of post-biopsy hemorrhage as in endomyocardial biopsy (52).

Finally, the cardiology panelists stated that there are also technical deficiencies in defining the pathological characterization of both endomyocardial and extracardiac biopsy samples in Turkey regarding evaluation sensitivity, limitations in the dye portfolio, and unavailability of mass spectrometric analysis of biopsy specimens.

The outputs of this panel highlighted that once CA is suspected, patients should be assessed by a multidisciplinary approach, where cardiology plays a central role in close collaboration with hematology, nuclear medicine, and radiology specialties, and patients should be referred to expert clinics at the earliest when and where the availability of essential diagnostic tools with experienced experts are lacking. In addition, the diagnostic approach in aTTR-CM should also be customized based on the availability of diagnostic tools/methods in each expert clinic to achieve a timely and definitive diagnosis.

## 5 Strengths and limitations

The consensuses reached in this panel are undoubtedly no further than the interpretations of available evidence and the current clinical environment in Turkey. The present study has all the limitations arising from the nature of the Delphi method (22, 23, 24). The absence of actual patient data, perspective, and experience within the discussions, the patient scenarios being discussed solely focused on aTTR-CM, the lack of consideration of differentiating characteristics of other amyloidosis subgroups, the different representation rates of specialties, the limited number of expert opinions, and the absence of experts from radiology, pathology, primary, and secondary healthcare providers all contribute to a limitation in accurately reflecting the diagnostic approaches in Turkey at an ideal level. In addition, while the panelists reached a consensus on the statement that all three radiotracers (99mTc-PYP, 99mTc-DPD, 99mTc-HMPD) can be used in scintigraphy imaging for the diagnosis of aTTR-CM, the majority of the experts had scintigraphy imaging experience only with 99mTc-PYP, which contradicted one of the objectives of the present study that aimed to reflect clinical practice in Turkey. However, this consensus statement, which is in line with the current evidence, was found to be essential to be mentioned in the study for the future availability of different radio tracers in Turkey. However, we did not assess nor discuss the score of the heart-to-whole body ratio for 99mTc-DPD and 99mTc-HDMP-based assessments, and this caused another limitation for this study.

Despite its limitations, we believe this study is important since it is the first expert opinion/consensus study in the current literature that investigates the diagnostic approaches of the physicians in CA by putting their own clinical setting in consideration. Even though the physicians who took part in this Delphi process were from experienced and advanced clinics in CA diagnosis in Turkey, they could still identify a substantial number of significant barriers to reach an accurate diagnosis in aTTR-CM. We believe these barriers are not exclusive to Turkey and are also present in most of the developed Western countries.

## 6 Future directions, implications for research and/or practice

A recent study in Turkey verified the considerable economic burden of HF in terms of both direct and indirect costs as well as underlined the importance of adopting improved prevention, management, and surveillance strategies (53). Therefore, prospective observational studies are needed to validate customized diagnostic algorithms for different clinical settings/capabilities. The current CA registry study in Turkey (PAPCAT) [with inclusion criteria of patients with 18 years of age or older, all patients with left ventricular hypertrophy (IVS ≥ 13 mm) without left ventricular pressure or volume overload, patients with hypertension or aortic stenosis who have an IVS of >15 mm, and patients with at least 2 red flag findings defined in a CA suspicion survey) can be a good platform to observe the impact of the recommendations provided in this study by comparing the accuracy of the previously established diagnosis (25). Innovative tools need to be developed and implemented to eliminate interpretation subjectivity while multidisciplinary collaboration will reduce errors and provide a timely diagnosis in aTTR-CM. The referral need for patients with suspected CA to expert clinics can be organized by implementing a hub-and-spoke model in Turkey. The hub-and-spoke model, as applied in healthcare settings, is a method of organization involving the establishment of a main campus or hub, which receives the heaviest resource investments and supplies the most intensive medical services, complemented by satellite campuses or spokes, which offer more limited-service arrays at sites distributed across the served market (54, 55). In this case, first of all, patient experience on their diagnostic journey including the interval observation between the appearance of symptoms and the time of diagnosis should be assessed (which can be done by conducting patient focus group meetings across multiple centers), and then standards should be established to determine the primary expert center or multiple expert centers in CA. Finally, referral rules should be implemented in both primary and secondary healthcare settings.

## Data Availability

Publicly available datasets were analyzed in this study. This is an expert consensus based study where all questions were answered via survey monkey and during a face-to-face discussion. Further enquiries can be directed to the corresponding author.
