# Supplementary material for: Current barriers and recommendations on the diagnosis of transthyretin amyloid cardiomyopathy: a Delphi study
Source: Front Cardiovasc Med. 2024 Jan 25;11:1299261. doi: 10.3389/fcvm.2024.1299261 (PMC10851939; doi:10.3389/fcvm.2024.1299261)

First-round Questions

**Dear Panelists,**

**This Delphi study aims to identify the uncertain areas in the non-invasive diagnosis journey of ATTR-CA by means of a multidisciplinary approach and develop relevant solution proposals. The first-round questions have been developed in the light of the international literature and based on the information and opinions obtained from the semi-structured interviews conducted with you.**

**The main subjects covered in this questionnaire are;**

- **Signs and symptoms that lead to a clinical suspicion,**
- **Methods used in the diagnostic journey and their role in clinical practice,**
- **And the adequacy of non-invasive methods in the diagnosis of ATTR-CA.**

**You are kindly requested to consider the document titled *“Diagnosis and treatment of cardiac amyloidosis: a position statement of the ESC Working Group on Myocardial and Pericardial Diseases”* that was published in 2021 when answering some of the questions in this questionnaire. An open access link to this publication has also been provided in related questions.**

**You are kindly requested not to leave blank the fields in open-ended questions in the case that they are related to your answer.**

**The questions in further rounds of this study will be developed by taking into account your answers to these questions together with your additional comments, and will be finalized to allow the development of recommendations for clinical practice in Turkey. In the 3rd round of the panel, which will be a face-to-face session, evaluation methods in the differential diagnosis of AL and interpretation of results will be discussed with hematology specialists.**

**With the consensus to be reached at the end of this study, it is also aimed to prepare an academic publication to reflect expert opinions that is supported by a systematic literature review.**

**Please feel free to contact us for any questions or requests for additional information.**

**Thank you for your contribution and support to this study, which we believe will lead to an important publication in this field.**

**Kind regards,**

* 1. Your name and surname:


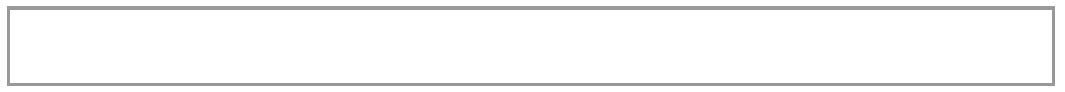


* 2. What is **the approximate number of patients** in whom you have suspected **cardiac amyloidosis (CA)** based on clinical signs and symptoms, ECG and ECHO findings **in the last 5 years**?

○ <10

○ 10-20

○ 21-40

○ 41-60

○ 61-80

○ ≥81

* 3. What is the approximate **proportion of patients** whom **you have** **diagnosed with CA** among those with suspected CA **in the last 5 years**?

○ <5%

○ 5-10%

○ 11-20%

○ 21-30%

○ 31-40%

○ ≥41%

* 4. What is the approximate **proportion of patients** whom **you have** **diagnosed with ATTR-CA among those** you **diagnosed with CA** in the last 5 years?

○ <5%

○ 5-10%

○ 11-20%

○ 21-30%

○ 31-40%

○ ≥41%

* 5. What is the approximate **proportion of patients** whom **you have** **diagnosed with light chain (AL)-CA among those** you **diagnosed with CA** in the last 5 years?

○ <5%

○ 5-10%

○ 11-20%

○ 21-30%

○ 31-40%

○ ≥41%

* 6. Please specify the diagnostic methods (**with corresponding % ratios**) you have used **in the last step** (before genetic testing) for the patients you have diagnosed with ATTR-CA in the last 5 years.

| Serum and urine tests for the differential diagnosis of AL amyloidosis | 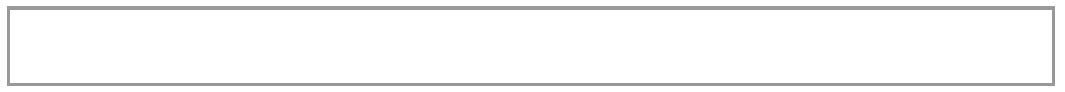 |
| --- | --- |
| Bone scintigraphy (PYP) | 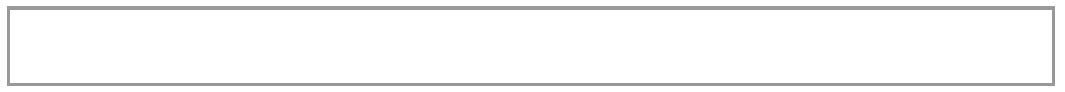 |
| Bone scintigraphy (MDP) | 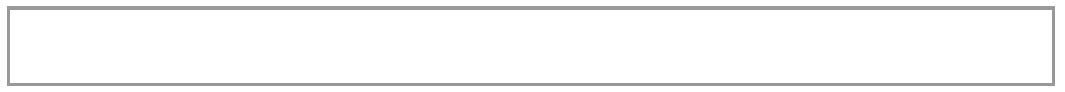 |
| Bone scintigraphy (DPD) | 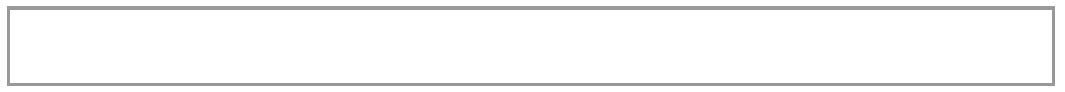 |
| Bone scintigraphy (HMDP) | 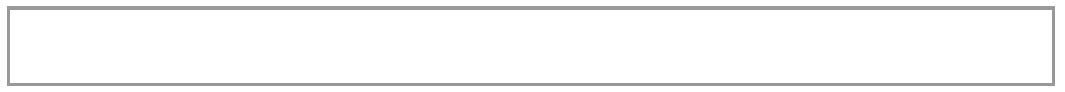 |
| Cardiac biopsy | 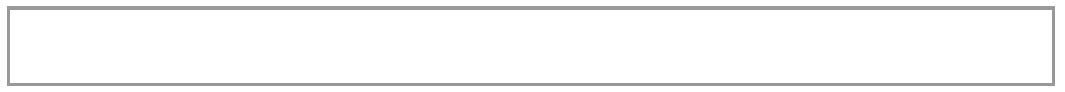 |
| Extra-cardiac biopsy | 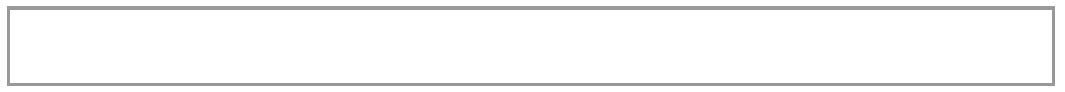 |
| Cardiac MR (CMR) | 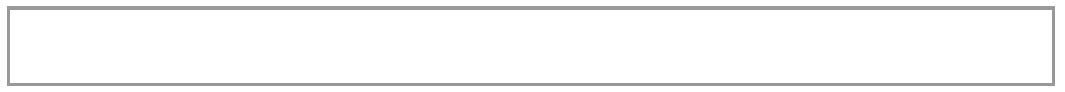 |

* 7. What is the proportion of patients you have diagnosed with ATTR-CA and also referred to genetic testing in the last 5 years?

○ All

○ None

○ Other;


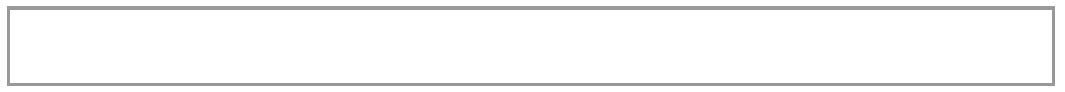


* 8. Do you think there is **an expected range concerning the age of onset** of clinical signs and symptoms of ATTR-CA among the patients in our country?

○ Yes

○ No

○ I am not sure

If your answer is ‘yes’, what are these age ranges in your opinion; and have you observed any difference between hereditary and wild-type cases?


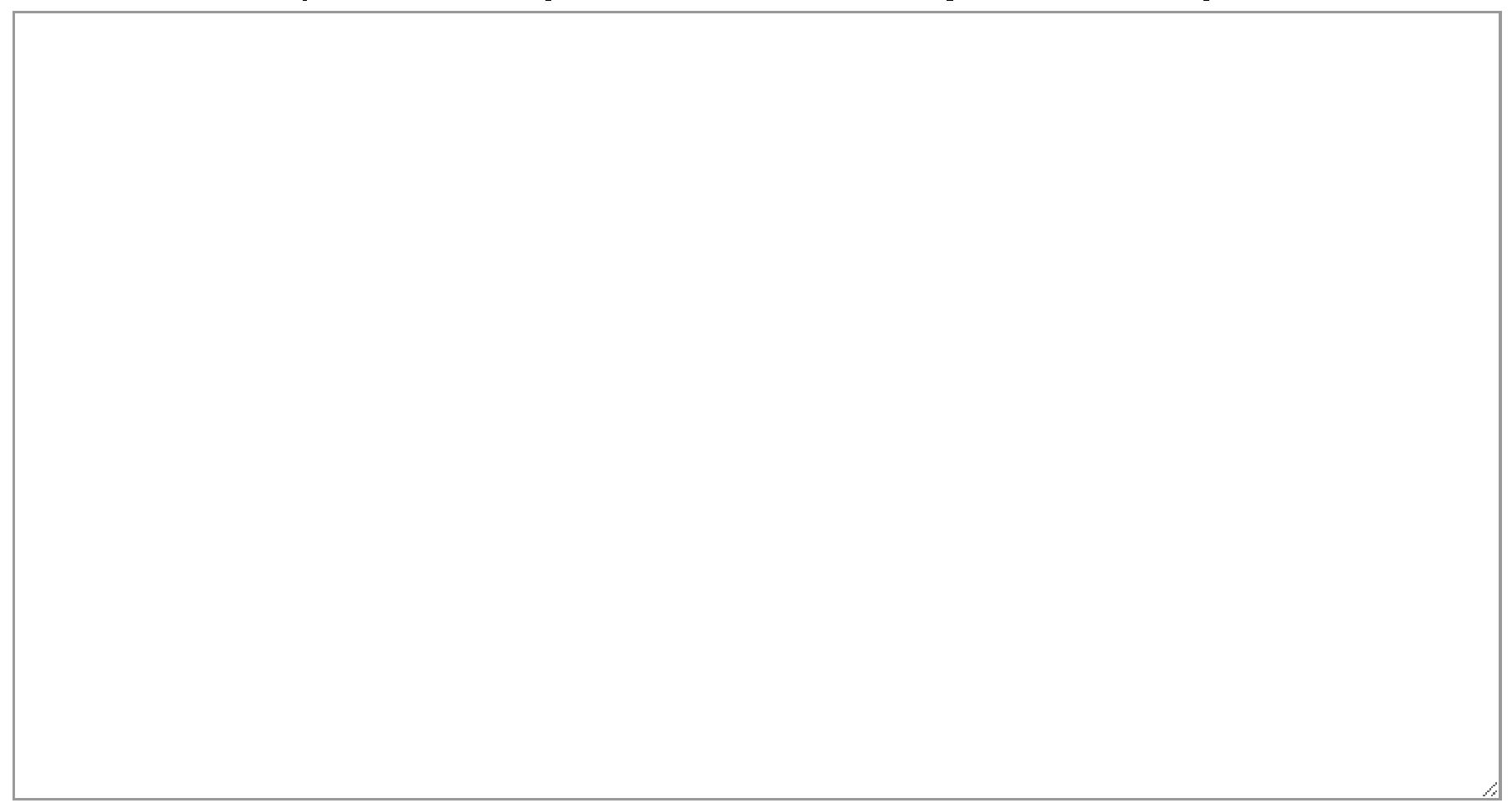


* 9. In our country, ATTR-CA prevalence is:

○ Higher in women

○ Higher in men

○ Equal in men and women in my opinion

○ I am not sure

10. If you think that the prevalence of ATTR-CA differs depending on gender in our country, should there be a gender-based difference in the diagnostic algorithm (differences in timing and sequence of methods used in the diagnostic pathway)?

○ Yes

○ No

If your answer to the question above is ‘yes’, what should be the differences in this diagnostic algorithm?


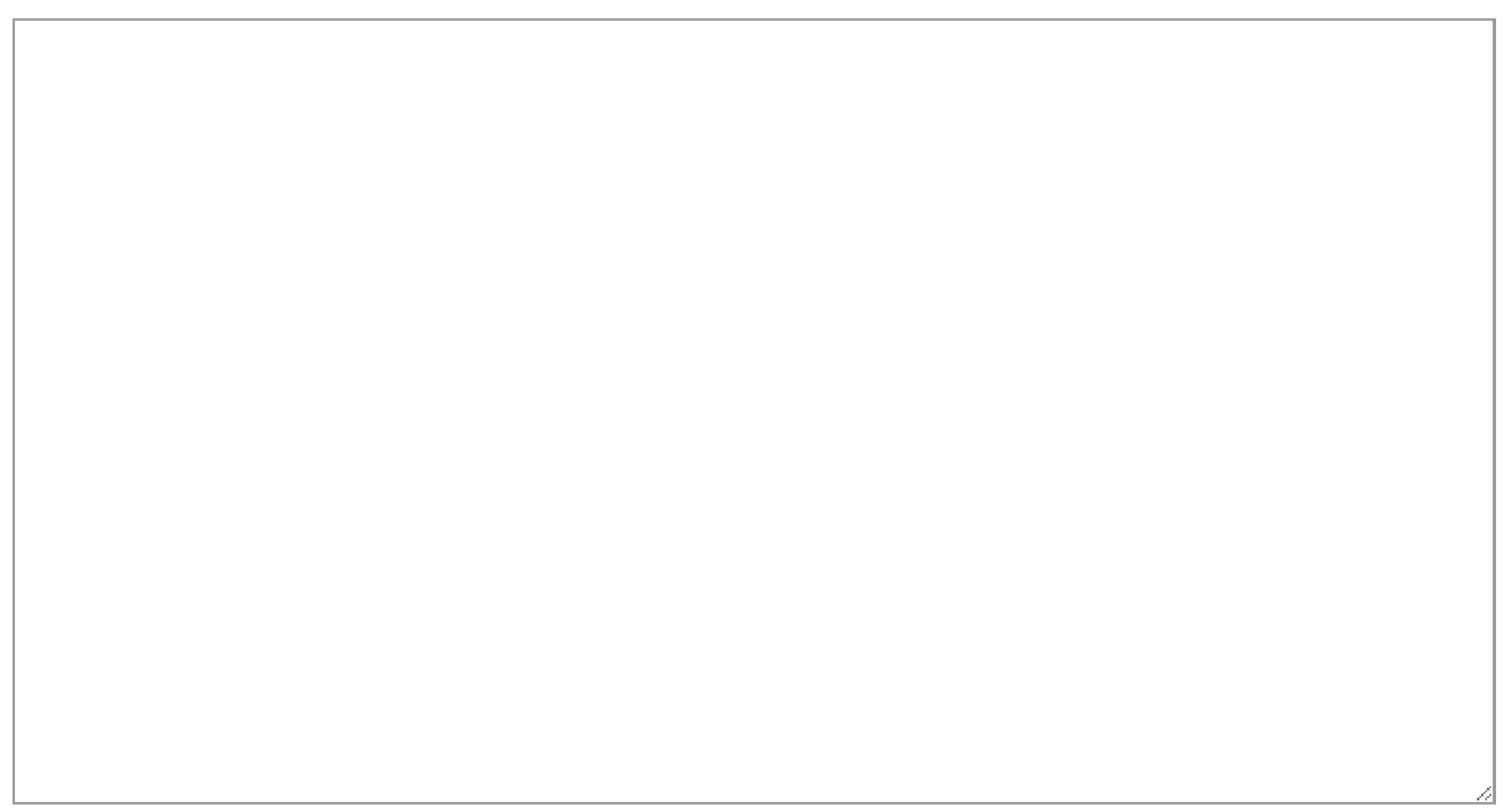


* 11. Please list, **in order of frequency**, the **cardiac** signs and symptoms you observe in patients diagnosed with ATTR-CA. (If there is any symptom in the following options that you have not encountered, you can **exclude them from the ranking by clicking the box on the right** for each option)

| 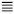 | 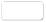 | Dyspnea | □ I have never encountered these symptoms |
| --- | --- | --- | --- |
| 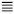 | 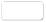 | Asthenia, fatigue | □ I have never encountered these symptoms |
| 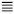 | 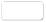 | Palpitations | □ I have never encountered these symptoms |
| 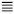 | 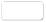 | Presyncope, syncope | □ I have never encountered these symptoms |
| 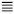 | 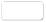 | Hypotension | □ I have never encountered these symptoms |
| 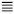 | 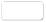 | Intolerance/resistance to antihypertensives | □ I have never encountered these symptoms |

12. **Apart from** the **cardiac signs and symptoms mentioned in the answers** to the question above, are there any **other cardiac** signs and symptoms that you encounter frequently, and if so, what are they?


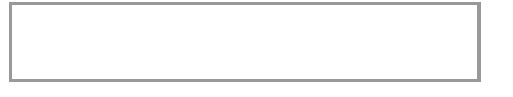


* 13. Please list, **in order of frequency**, the **extra-cardiac** symptoms you observe in patients diagnosed with ATTR-CA. (If there is any symptom in the following options that you have not encountered, you can **exclude them from the ranking by clicking the box on the right** for each option)

| 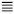 | 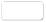 | Renal findings (proteinuria, renal insufficiency) | □ I have never encountered these symptoms |
| --- | --- | --- | --- |
| 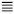 | 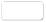 | Central nervous system findings (dementia, headache, seizure, stroke) | □ I have never encountered these symptoms |
| 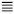 | 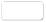 | Peripheral nervous system findings (peripheral sensory-motor neuropathy) | □ I have never encountered these symptoms |
| 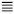 | 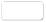 | Autonomic nervous system findings (orthostatic hypotension, urinary retention) | □ I have never encountered these symptoms |
| 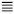 | 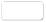 | Soft tissue findings (macroglossia, periorbital purpura, subcutaneous amyloidoma) | □ I have never encountered these symptoms |
| 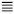 | 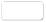 | Ocular findings | □ I have never encountered these symptoms |
| 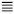 | 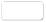 | Bilateral carpal tunnel syndrome | □ I have never encountered these symptoms |
| 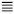 | 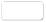 | Hepatomegaly, ascites | □ I have never encountered these symptoms |
| 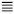 | 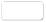 | Diarrhea | □ I have never encountered these symptoms |
| 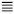 | 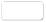 | Family history of CA | □ I have never encountered these symptoms |

14. **Apart from** the **extra-cardiac signs and symptoms mentioned in the answers** to the question above, are there any **other extra-cardiac** signs and symptoms that you encounter frequently, and if so, what are they?


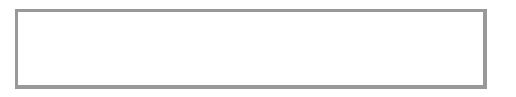


* 15. What is the proportion of patients diagnosed with ATTR-CA in whom you have observed **elevated NT-proBNP or persistently high troponin levels disproportionate to the degree of heart failure**?

○ I have never encountered such results

○ I do not have sufficient clinical experience to estimate this proportion

○ <10%

○ 10%-20%

○ 21%-30%

○ 31%-40%

○ 41%-50%

○ >50%

* 16. What are **the 3 most common ECG findings you observe** in your patients diagnosed with ATTR-CA?

□ Normal ECG findings despite left ventricular hypertrophy

□ Low QRS voltage despite left ventricular hypertrophy

□ Pseudo-infarct findings

□ Decreased R progression in chest leads

□ AV block

□ Branch block

□ AF

□ Other;


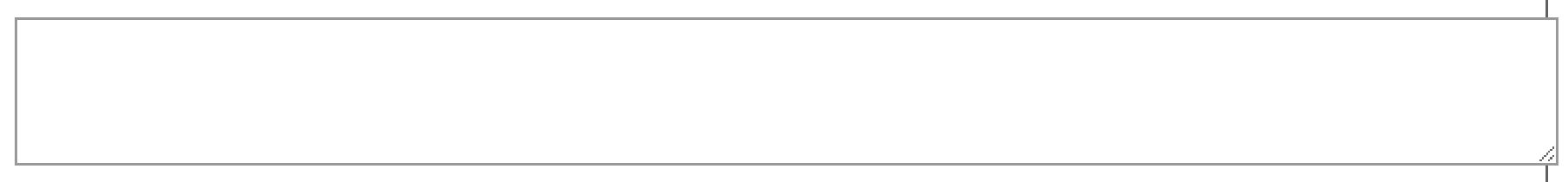


* 17. What is **the proportion of patients** with LV thickness **greater than** 12mm on ECHO among those **you have diagnosed** with ATTR-CA?

○ I have never encountered such results

○ I do not have sufficient clinical experience to estimate this proportion

○ <10%

○ 10%-20%

○ 21%-30%

○ 31%-40%

○ 41%-50%

○ >50%

* 18. What are **the 3 most common ECHO findings, apart from left ventricular hypertrophy**, you see in patients you diagnose with ATTR-CA?

□ Biventricular hypertrophy

□ Isolated right ventricular hypertrophy

□ Decreased right ventricular function, right ventricular failure

□ Biatrial dilatation

□ Isolated left atrial dilatation

□ Isolated right atrial dilatation

□ Diastolic dysfunction

□ Granular sparkling pattern in the ventricle

□ Decreased longitudinal strain with apical sparing pattern

□ Increased AV valve thickness

□ Pericardial effusion

□ Other


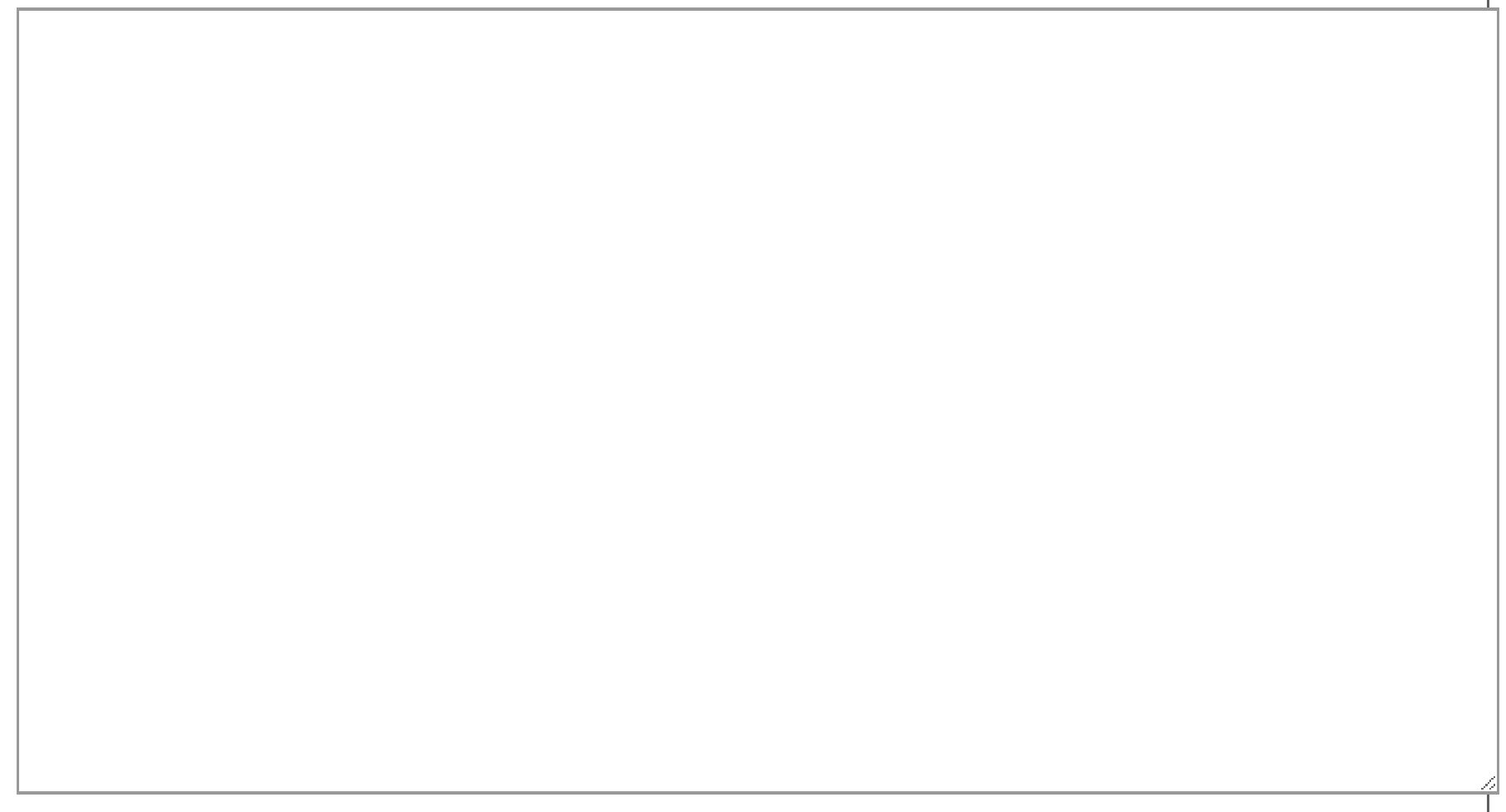


* 19. The document titled *“Diagnosis and treatment of cardiac amyloidosis: a position statement of the ESC Working Group on Myocardial and Pericardial Diseases”* and published in 2021 is [**consistent** with my clinical experience/opinions](https://onlinelibrary.wiley.com/doi/epdf/10.1002/ejhf.2140) **in terms of the association of clinical signs and symptoms that may indicate suspected CA**. [Click here to access the related ESC publication](https://onlinelibrary.wiley.com/doi/epdf/10.1002/ejhf.2140) and to check Figure 3, which presents the conditions suggestive of a clinical suspicion.

○ Strongly agree

○ Agree

○ Undecided

○ Disagree

○ Strongly disagree

20. If your answer to the question above is **‘undecided’ or ‘disagree’**, please specify the reason (if there are **parts** of the relevant ESC publication **that differ from your clinical observation and approach** in terms of clinical suspicion, please specify them as well).


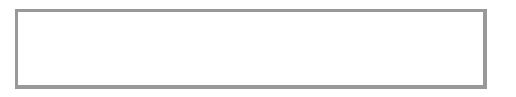


* 21. In case of clinical suspicion based on the **red flags** stated in the above ESC publication, further investigations should be planned for the differential diagnosis of CA.

○ Strongly agree

○ Agree

○ Undecided

○ Disagree

○ Strongly disagree

22. If your answer to the question above is **‘undecided’ or ‘disagree’**, please specify the reason.


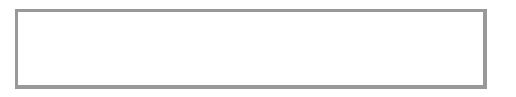


* 23. **In case of clinical suspicion** based on the above ESC publication, **if it is not possible** to perform and evaluate further investigations for the differential diagnosis of CA in the institution, **a referral to an institution that is competent in this field** should be made as soon as possible.

○ Strongly agree

○ Agree

○ Undecided

○ Disagree

○ Strongly disagree

24. If your answer to the question above is **‘undecided’ or ‘disagree’**, please specify the reason.


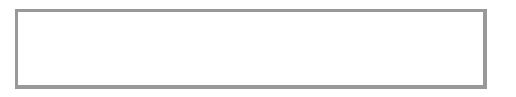


* 25. **Which of the following tests (and in which order)** do you usually perform in order to rule out other CA types and establish **a diagnosis of ATTR-CA** in a patient with **suspected CA based on clinical, ECG and ECHO findings**? (Please specify the order in line with **your own clinical capabilities and practice**; you can exclude the methods you do not use from the ranking by clicking the box on the right for each option)

| 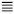 | 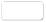 | Serum and urine tests for the differential diagnosis of AL amyloidosis (serum free light chain (sFLC) test, serum (SPIE) and urine (UPIE) protein immunofixation electrophoresis) | □ I do not use this method |
| --- | --- | --- | --- |
| 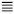 | 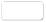 | Bone scintigraphy | □ I do not use this method |
| 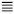 | 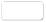 | Cardiac biopsy | □ I do not use this method |
| 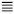 | 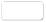 | Extra-cardiac biopsy | □ I do not use this method |
| 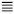 | 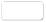 | Cardiac MR (CMR) | □ I do not use this method |
| 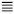 | 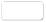 | Genetic testing | □ I do not use this method |

26. Are there any comments you would like to add regarding the order you have specified in the question above?


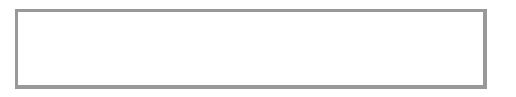


* 27. Which lab tests do you **routinely** request in your clinic for the differential diagnosis of AL amyloidosis? (You may select multiple options)

□ Serum free light chain test (sFLC assay)

□ Serum protein immunofixation electrophoresis (SPIE)

□ Urine immunofixation electrophoresis (UPIE)

□ Other:


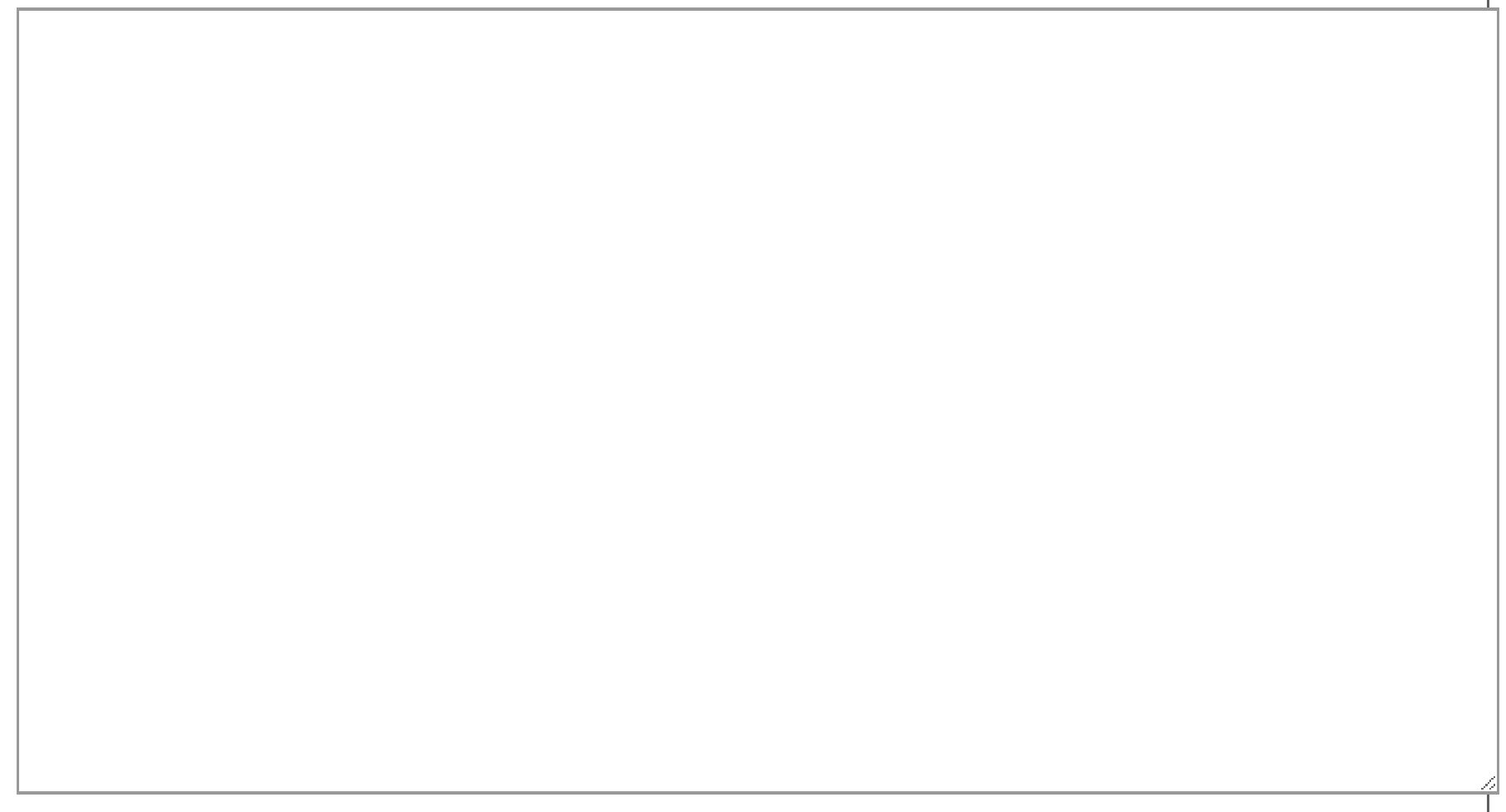


* 28. In a patient whose **clinical, ECG and ECHO findings are suggestive of CA** and **lab tests** (sFLC, SPIE, UPIE) **are** found to be **against AL***, which diagnostic method would you prefer to use **in the next step**? (If you have simultaneous preferences for the next step, [you can choose more than one option) Click here to access](https://onlinelibrary.wiley.com/doi/epdf/10.1002/ejhf.2140) the tests for the differential diagnosis of AL and result interpretation approaches (Table 3) in the aforementioned ESC publication.

□ I would refer the patient to bone scintigraphy.

□ I would refer the patient to cardiac MRI.

□ I would refer the patient to cardiac biopsy.

□ I would refer the patient to extra-cardiac biopsy.

□ Other:


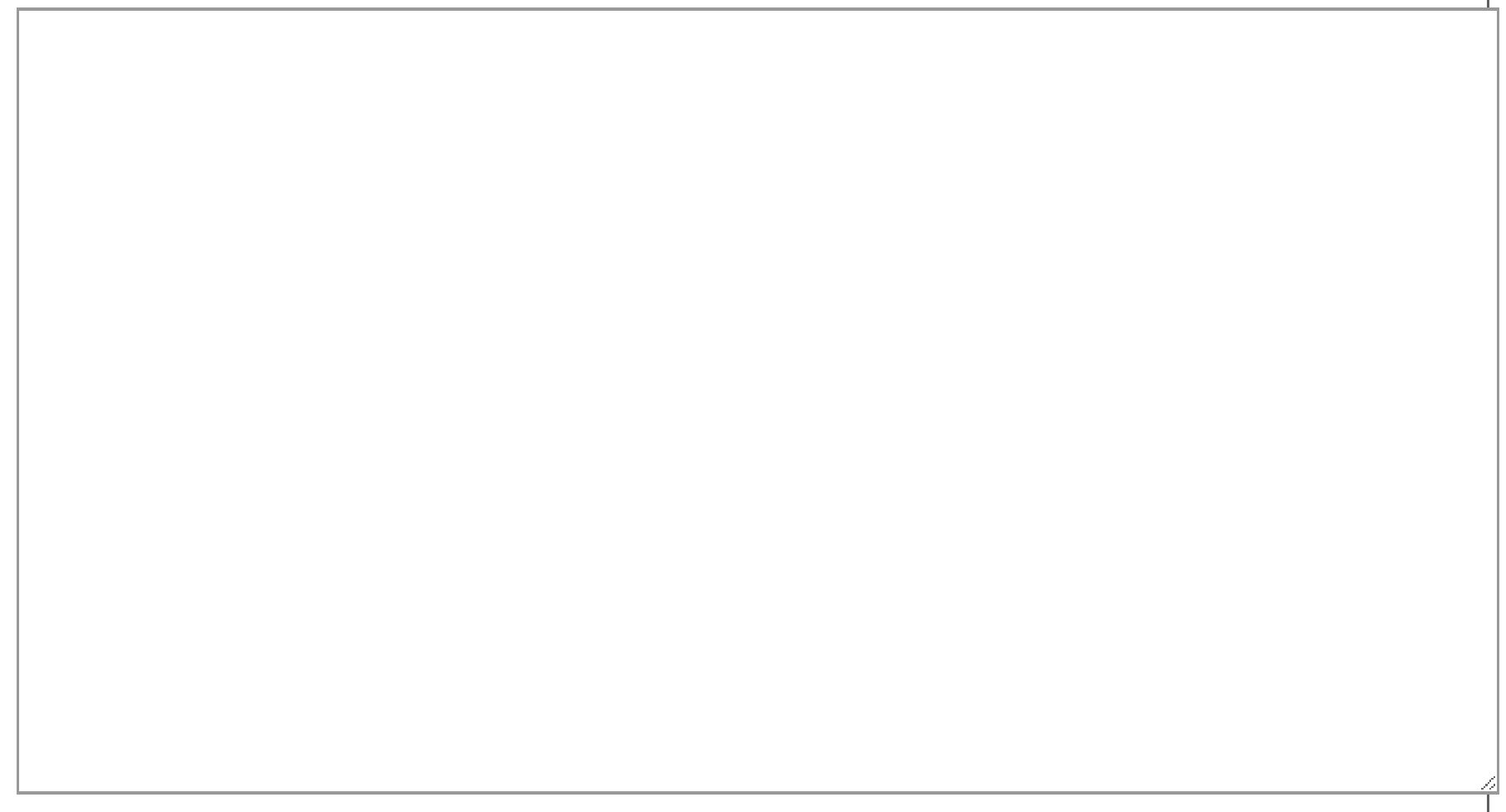


* 29. In a patient with **suspected CA** whose **lab tests** (sFLC, SPIE, UPIE) are found to be **in favor of AL***, which diagnostic method would you prefer to use **in the next step**? (If you have simultaneous preferences for the next step, you can choose more than one option)

□ I would refer the patient to bone scintigraphy.

□ I would refer the patient to cardiac MRI.

□ I would refer the patient to cardiac biopsy.

□ I would refer the patient to extra-cardiac biopsy.

□ I would refer the patient to the hematology department for a more detailed evaluation with a provisional diagnosis of AL amyloidosis.

□ Other


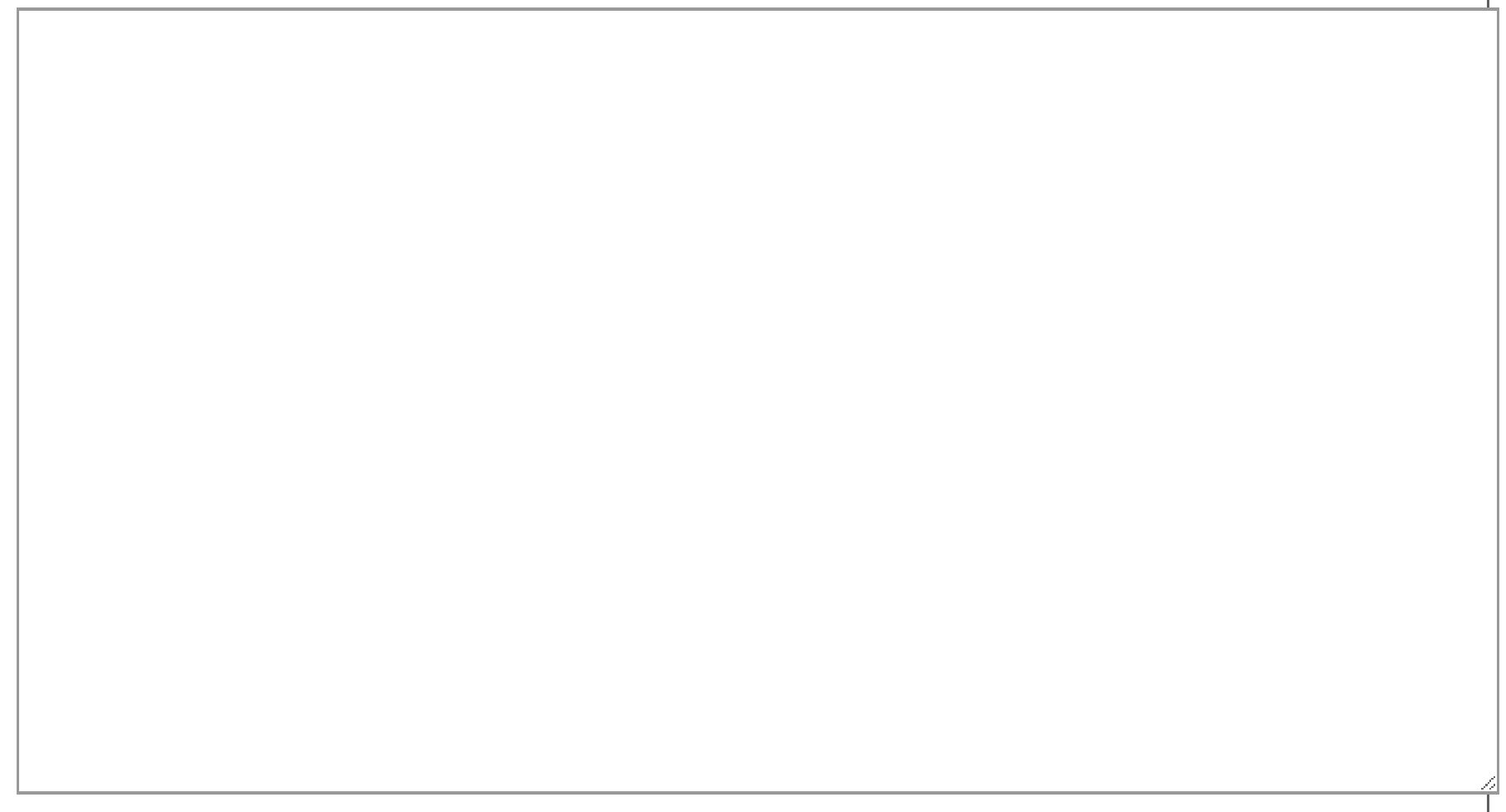


* 30. **If the lab tests** performed for suspected AL (sFLC,SPIE, UPIE) provide results that **are against AL**, then **AL amyloidosis can be certainly ruled out**.

○ Strongly agree

○ Agree

○ Undecided

○ Disagree

○ Strongly disagree

31. If your answer to the question above is **‘undecided’ or ‘disagree’**, please specify the reason.


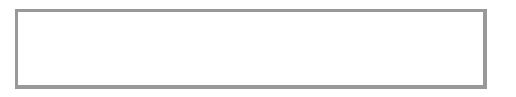


* 32. **For** a patient whose **lab tests** (sFLC, SPIE, UPIE) performed due to suspected AL are found to be **against AL,** **a diagnosis of ATTR may often be established in the presence of ATTR-specific scintigraphy findings** **(Grade ≥2, H/CL ≥1.5)**.

○ Strongly agree

○ Agree

○ Undecided

○ Disagree

○ Strongly disagree

33. If your answer to the question above is **‘undecided’ or ‘disagree’**, please specify the reason.


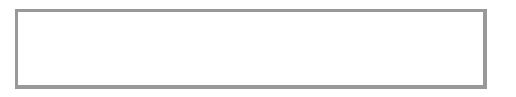


Explanation for the questions;

**For the questions from #34 to #39, please answer according to the clinical scenario provided below, assuming that scintigraphy was performed using 99mTc-PYP.**

- **A patient whose clinical signs, symptoms, ECG, ECHO and/or cardiac MRI are suggestive of CA (with ECHO and MRI already performed)**
- **Lab tests (where sFLC, SPIE, UPIE are all completed) are found to be against AL;**

* 34. Which of the following options/combination of options best describes **your approach to ATTR diagnosis** in this patient in the presence of **Grade 3 uptake H/CL ≥1.5 (at 1 hour)** observed by **planar** scintigraphy imaging alone? (Please choose your answers **in line with your current clinical capabilities and your own preferences and not based on ideal conditions**. Multiple options may be selected)

□ I would diagnose ATTR based on these findings without any further investigation.

□ I would diagnose ATTR if additional SPECT imaging confirms the consistency with ATTR in addition to the these findings.

□ I would diagnose ATTR if additional SPECT-CT imaging confirms the consistency with ATTR in addition to the these findings.

□ In addition to these findings, if there is excessive blood pool activity in the imaging at 1 hour, I would definitely request imaging at 3 hours as well.

□ I would still request cardiac biopsy, even if all additional imaging tests I choose reveal results consistent with ATTR in this scenario.

□ I would request a biopsy in the case of discordant additional results in this scenario.

□ I would certainly review the CMR findings once more.

□ None of the above

□ My alternative approach to diagnosis and associated reasons in this scenario;


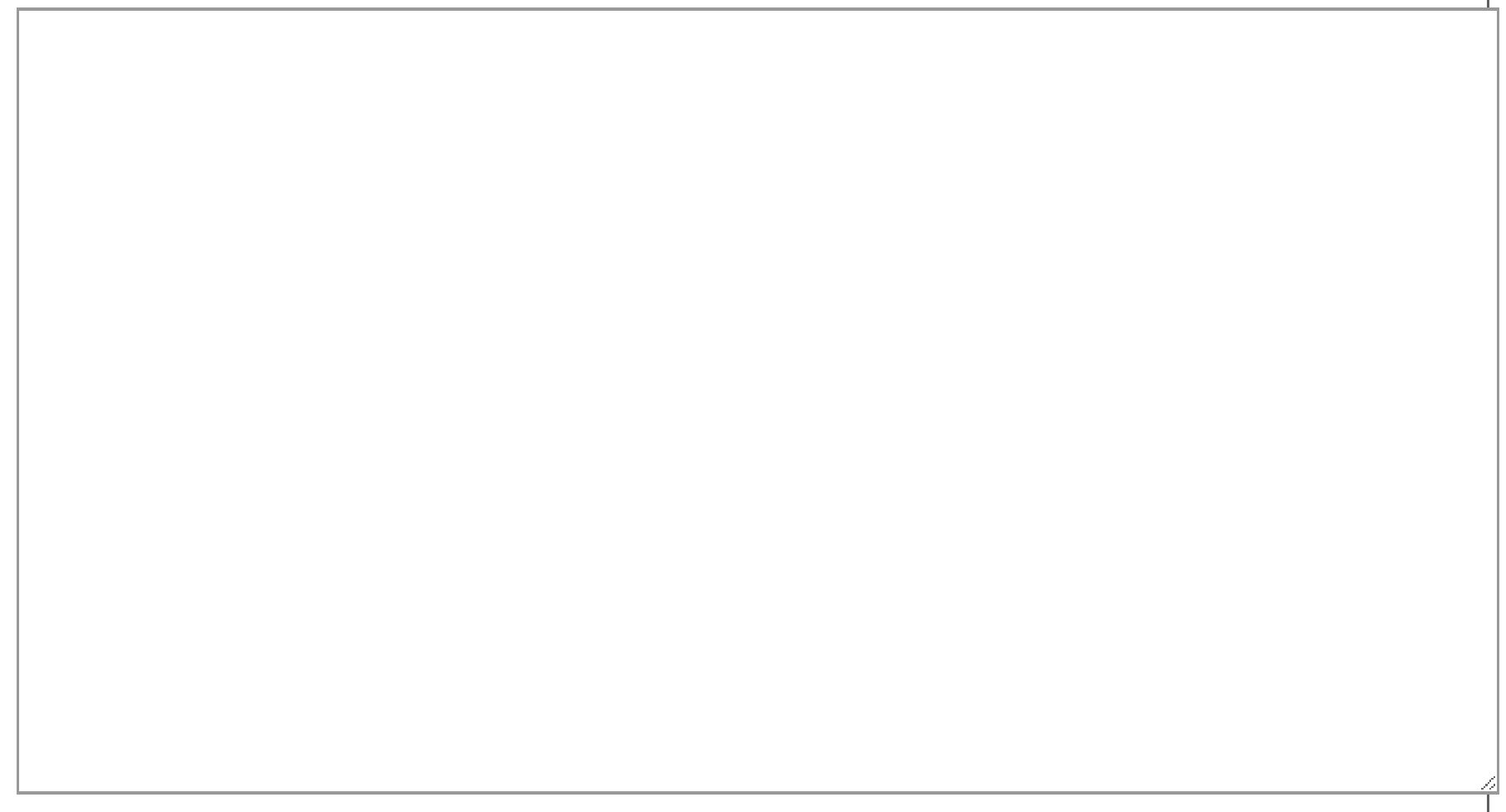


* 35. Which of the following options/combination of options best describes **your approach to ATTR diagnosis** in this patient in the presence of **Grade 3 uptake H/CL 1.5< (at 1 hour)** observed by **planar** scintigraphy imaging alone? (Please choose your answers **in line with your current clinical capabilities and your own preferences** and not based on ideal conditions. Multiple options may be selected)

□ I would diagnose ATTR based on these findings without any further investigation.

□ I would diagnose ATTR if additional SPECT imaging confirms the consistency with ATTR (H/CL ≥1.5) in addition to the these findings.

□ I would diagnose ATTR if additional SPECT-CT imaging confirms the consistency with ATTR (H/CL ≥1.5) in addition to the these findings.

□ In addition to these findings, if there is excessive blood pool activity in the imaging at 1 hour, I would definitely request imaging at 3 hours as well.

□ I would review the CMR findings once more.

□ I would still request cardiac biopsy, even if all additional imaging tests I choose reveal results consistent with ATTR in this scenario.

□ I would request a biopsy in the case of discordant results in addition to the initial results in this scenario.

□ None of the above

□ My alternative approach to diagnosis and associated reasons in this scenario;


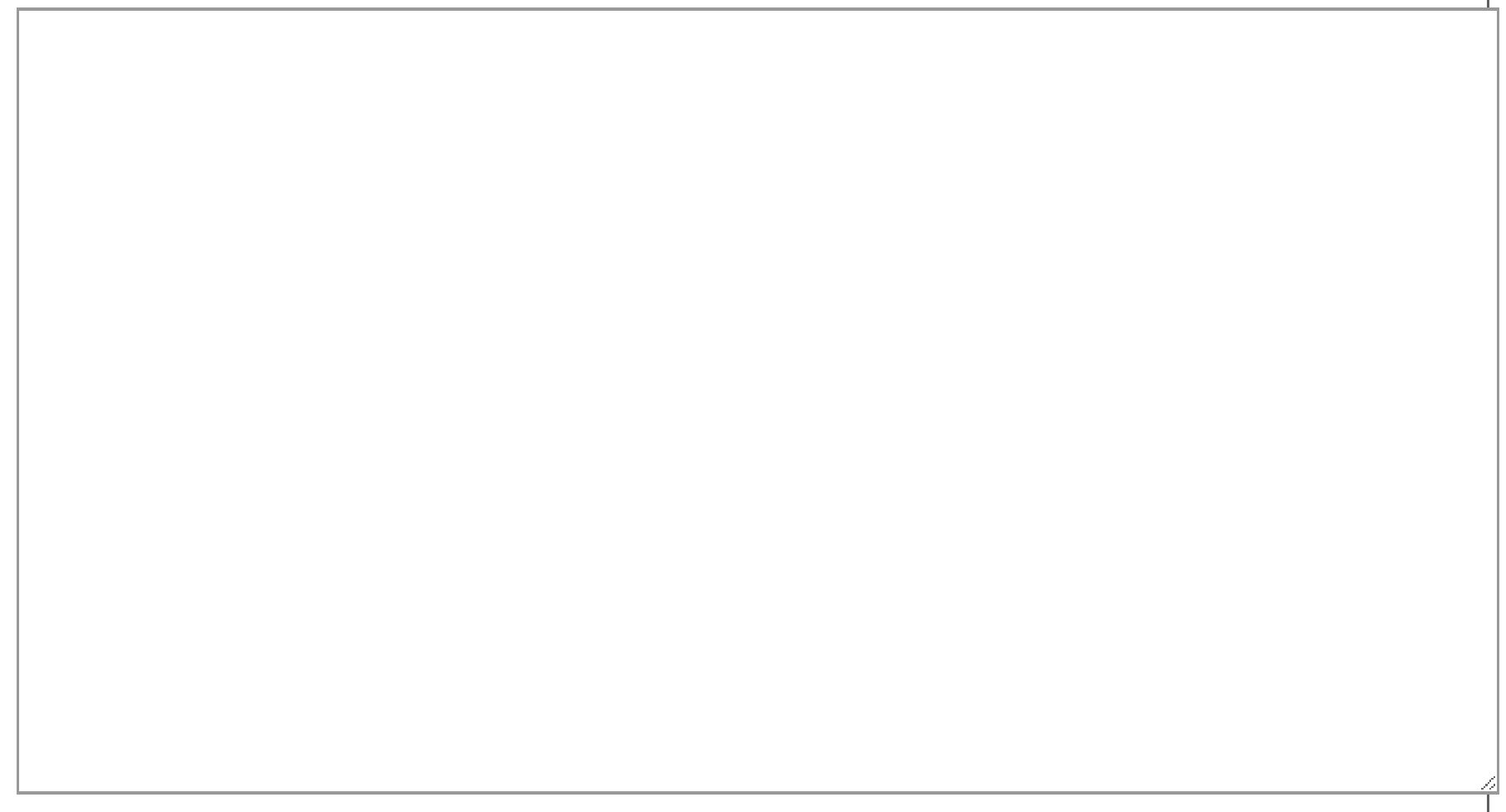


* 36. Which of the following options or combination of options best describes **your approach to ATTR diagnosis** in this patient in the presence of **Grade 2 uptake, H/CL ≥1.5 (at 1 hour)** observed by **planar** scintigraphy imaging alone? (Please choose your answers in line with your current clinical capabilities and your own preferences and not based on ideal conditions. You may choose more than one option; please pay attention to the consistency of the options you choose)

□ I would diagnose ATTR based on these findings without any further investigation.

□ I would diagnose ATTR if additional SPECT imaging confirms the consistency with ATTR in addition to the these findings.

□ I would diagnose ATTR if additional SPECT-CT imaging confirms the consistency with ATTR in addition to the these findings.

□ In addition to these findings, if there is excessive blood pool activity in the imaging at 1 hour, I would definitely request imaging at 3 hours as well.

□ I would review the CMR findings once more.

□ I would still request cardiac biopsy, even if all additional imaging tests I choose reveal results consistent with ATTR in this scenario.

□ I would request a biopsy in the case of additional discordant results in this scenario.

□ None of the above

□ My alternative approach to diagnosis and associated reasons in this scenario;


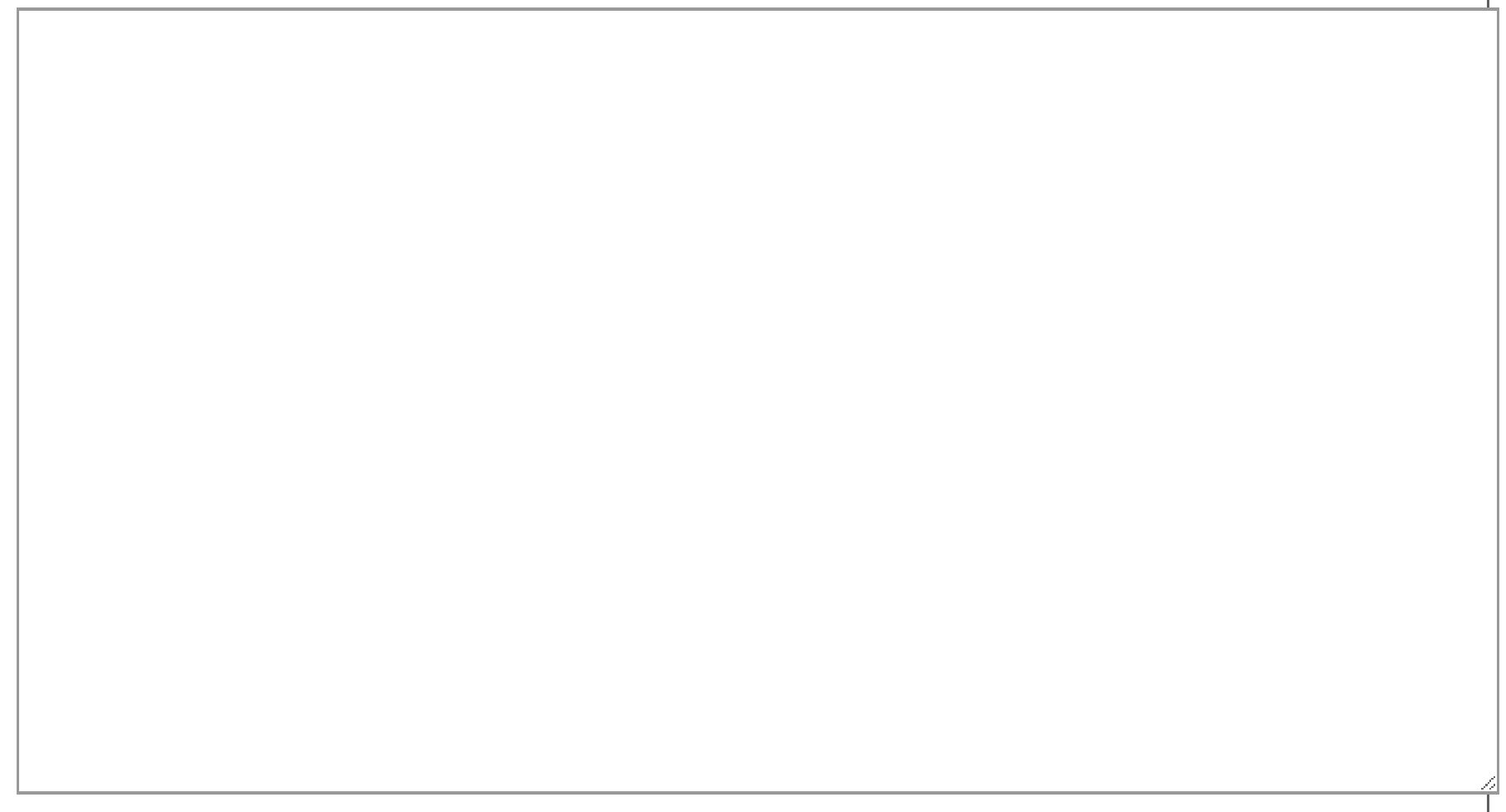


* 37. Which of the following options or combination of options best describes your approach to ATTR diagnosis in this patient in the presence of **Grade 2 uptake, H/CL 1.5< (at 1 hour)** observed **by** **planar** scintigraphy imaging **alone**? (Please choose your answers in line with your current clinical capabilities and your own preferences and not based on ideal conditions. Multiple options may be selected)

□ I would diagnose ATTR based on these findings without any further investigation.

□ I would diagnose ATTR if additional SPECT imaging confirms the consistency with ATTR (H/CL >1.5) in addition to the these findings.

□ I would diagnose ATTR if additional SPECT-CT imaging confirms the consistency with ATTR (H/CL >1.5) in addition to the these findings.

□ In addition to these findings, if there is excessive blood pool activity in the imaging at 1 hour, I would definitely request imaging at 3 hours as well.

□ I would review the CMR findings once more.

□ I would still request cardiac biopsy, even if all additional imaging tests I choose reveal results consistent with ATTR in this scenario.

□ I would request a biopsy only in the case of discordant additional results in this scenario.

□ None of the above

□ My alternative approach to diagnosis and associated reasons in this scenario;


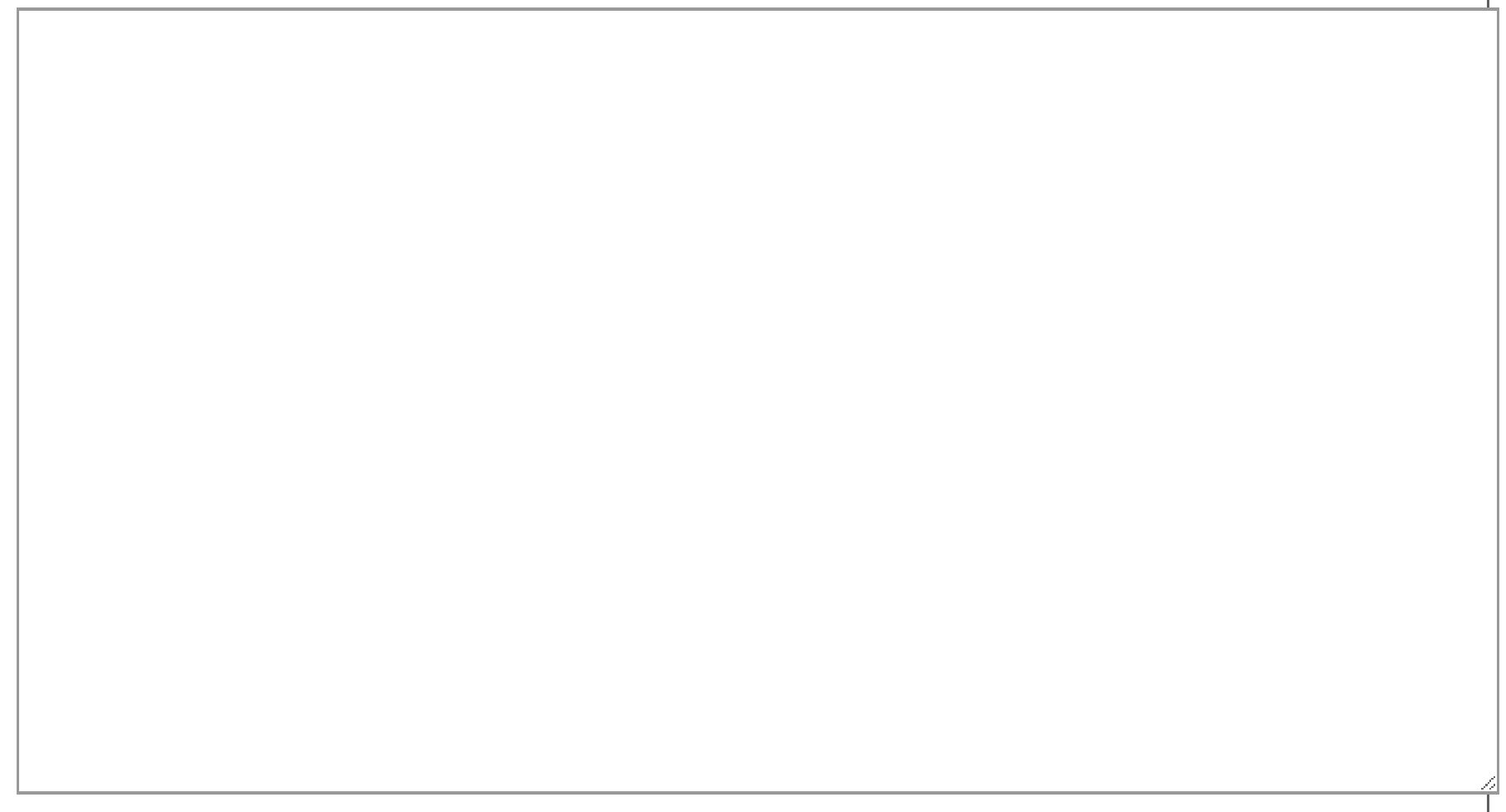


* 38. Which of the following options or combination of options best describes your approach to ATTR diagnosis in this patient in the presence of **Grade 1 uptake, H/CL ≥1.5 (at 1 hour)** observed **by** **planar** scintigraphy imaging **alone**? (Please choose your answers in line with your current clinical capabilities and your own preferences and not based on ideal conditions. Multiple options may be selected)

□ I would likely diagnose ATTR based on these findings without any further investigation.

□ I would diagnose ATTR if additional SPECT imaging confirms the consistency with ATTR (Grade ≥2 uptake) in addition to the these findings.

□ I would diagnose ATTR if additional SPECT-CT imaging confirms the consistency with ATTR (Grade ≥2 uptake) in addition to the these findings.

□ In addition to these findings, if there is excessive blood pool activity in the imaging at 1 hour, I would definitely request imaging at 3 hours as well.

□ I would review the CMR findings once more.

□ I would still request cardiac biopsy, even if all additional imaging tests I choose reveal results consistent with ATTR in this scenario.

□ I would request a biopsy only in the case of discordant additional results in this scenario.

□ None of the above

□ My alternative approach to diagnosis and associated reasons in this scenario;


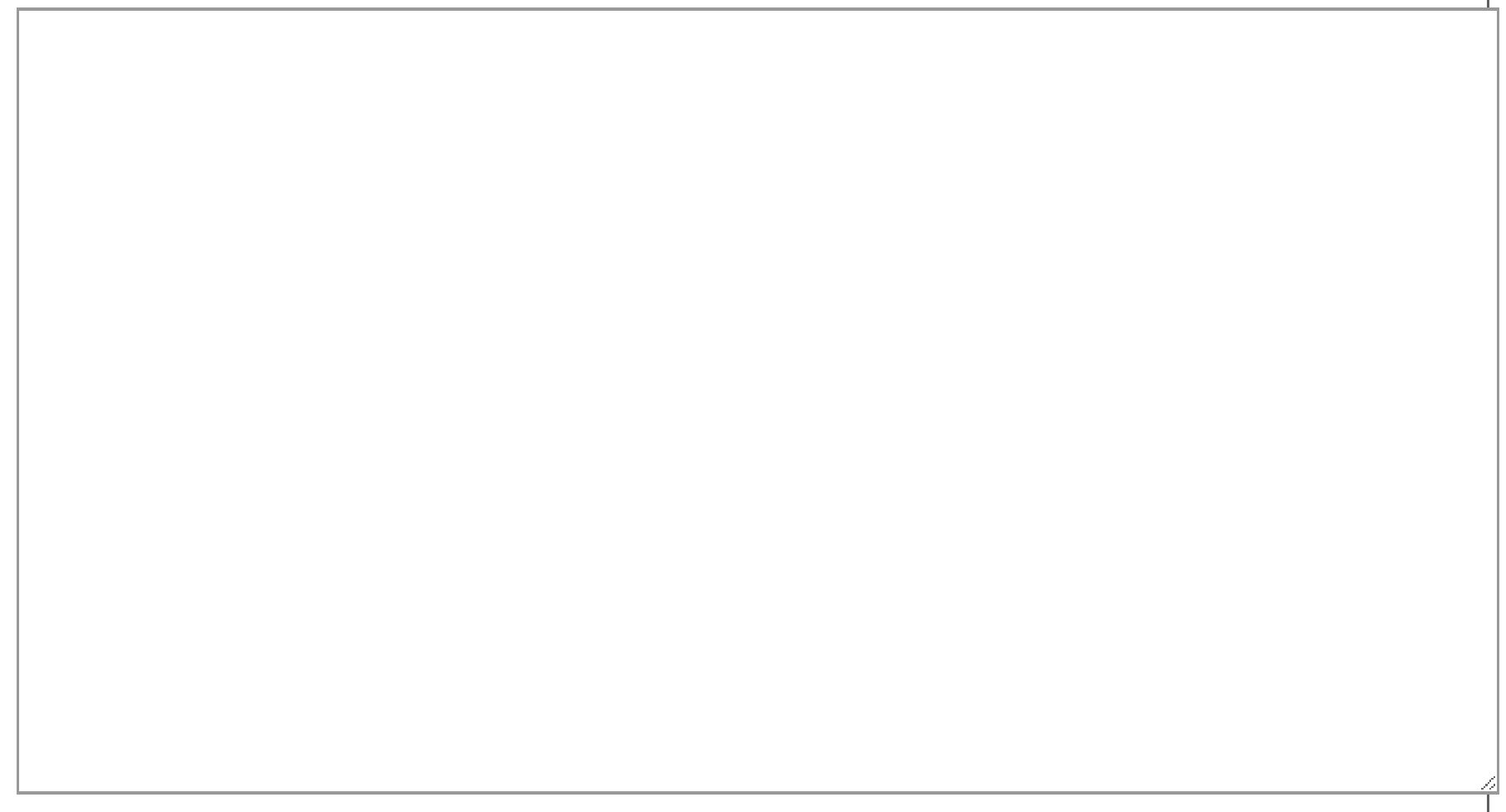


* 39. Which of the following options or combination of options best describes your approach to ATTR diagnosis in this patient in the presence of **Grade 1 uptake, H/CL 1.5< (at 1 hour)** observed **by** **planar** scintigraphy imaging **alone**? (Please choose your answers in line with your current clinical capabilities and your own preferences and not based on ideal conditions. Multiple options may be selected)

□ I would likely diagnose ATTR based on these findings without any further investigation.

□ I would diagnose ATTR if additional SPECT imaging confirms the consistency with ATTR (Grade ≥2, H/CL ≥1.5) in addition to the these findings.

□ I would diagnose ATTR if additional SPECT-CT imaging confirms the consistency with ATTR (Grade ≥2, H/CL ≥1.5) in addition to the these findings.

□ In addition to these findings, if there is excessive blood pool activity in the imaging at 1 hour, I would definitely request imaging at 3 hours as well.

□ I would review the CMR findings once more.

□ I would still request cardiac biopsy, even if all additional imaging tests I choose reveal results consistent with ATTR in this scenario.

□ I would request a biopsy only in the case of discordant additional results in this scenario.

□ None of the above

□ My alternative approach to diagnosis and associated reasons in this scenario;


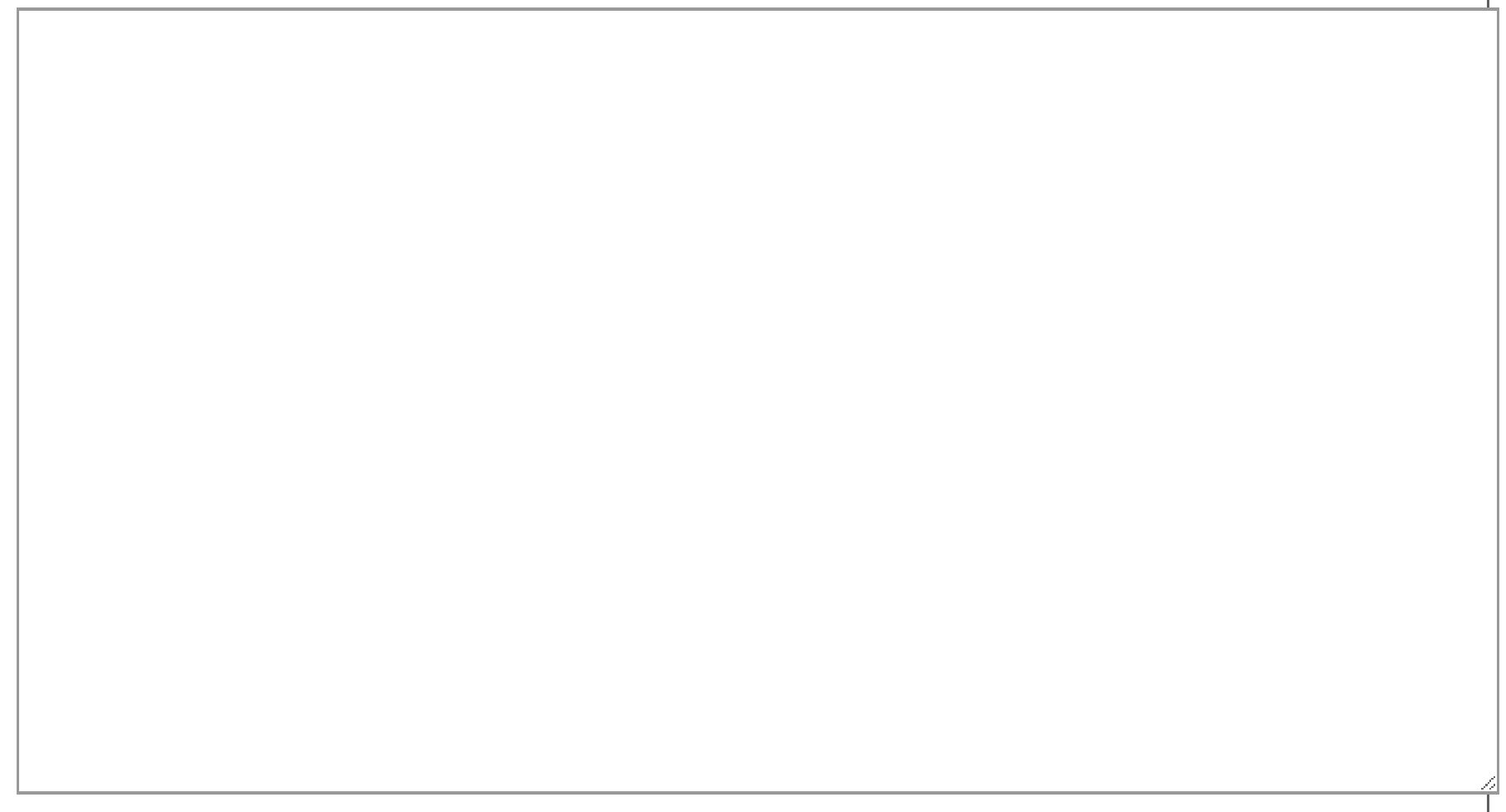


* 40. What is **the approximate proportion of false positive results** for ATTR-CA **you encounter** in bone scintigraphy investigations performed for CA?

○ I have never encountered such results.

○ I do not have sufficient clinical experience to estimate this proportion.

○ <5%

○ 6%-10%

○ 11%-20%

○ 21%-35%

○ >35%

* 41. **If you have encountered false positivity** for ATTR-CA in bone scintigraphy, could you please specify **the special conditions of your patients** that caused this situation? (Multiple options may be selected)

□ AL amyloidosis

□ Blood pool uptake (planar imaging)

□ Rib fracture (planar imaging)

□ Valvular/annular calcifications

□ Myocardial infarction (acute or subacute MI)

□ Hydroxychloroquine cardiotoxicity

□ Other rare types of cardiac amyloidosis

□ I have encountered false positivity but the exact cause could not be identified.

□ I have never encountered false positivity

□ Other


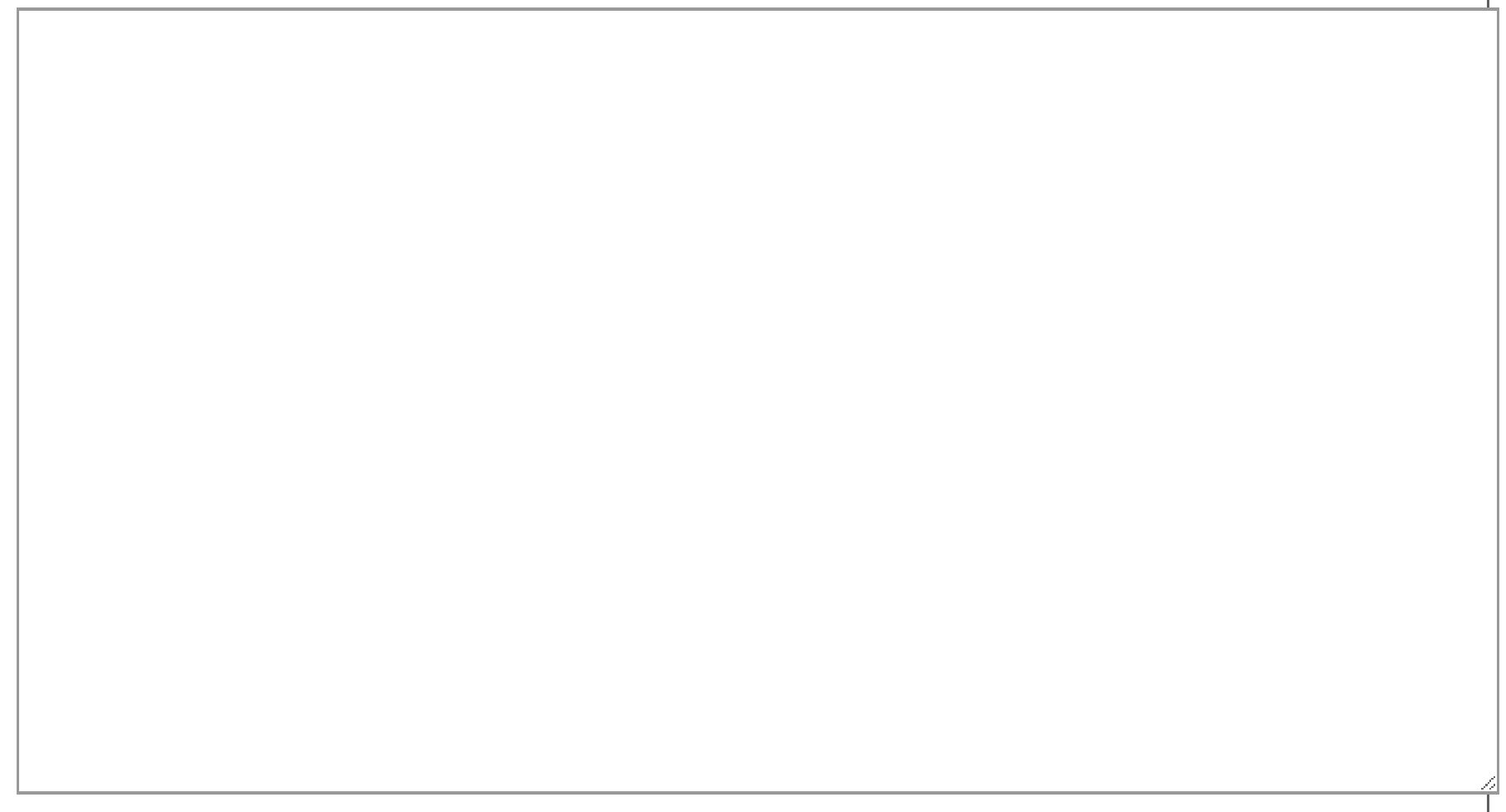


42. Could you please summarize the additional approaches you apply in order to establish the diagnosis in cases where you encounter false positive results?


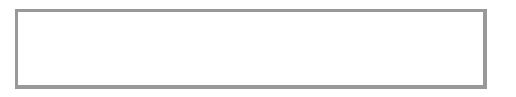


* 43. What is **the approximate proportion of false negative results** for ATTR-CA **you encounter** in bone scintigraphy investigations performed for CA?

○ I have never encountered such results.

○ I do not have sufficient clinical experience to estimate this proportion.

○ <5%

○ 6%-10%

○ 11%-20%

○ 21%-35%

○ >35%

* 44. **If you have encountered false negativity for ATTR-CA** in bone scintigraphy, could you please specify **the special conditions of your patients** that caused this situation?

□ Early-stage disease

□ Myocardial scarring resulting from long past MI

□ Delayed uptake of radioactive agent (Delayed acquisition)

□ Early uptake of radioactive agent (Premature acquisition)

□ Certain TTR mutation subtypes

□ I have encountered false negativity but the exact cause could not be identified.

□ I have never encountered false negativity.

□ Other


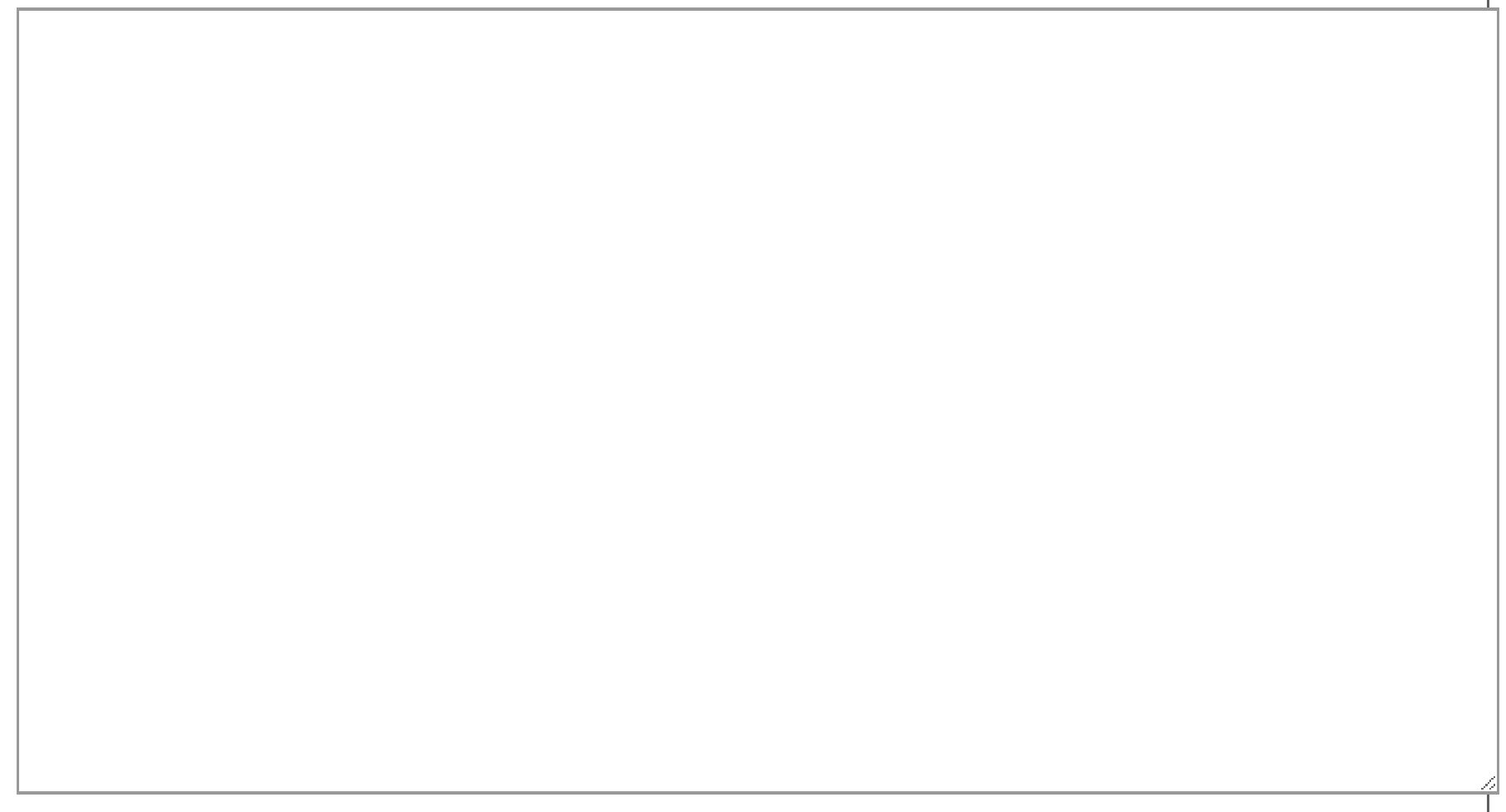


45. Could you please summarize the additional approaches you apply in order to establish the diagnosis in cases where you encounter false negative results?


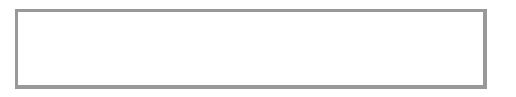


* 46. Could you please specify the actions that can substantially/significantly **reduce the errors caused by non-invasive diagnostic methods** in the diagnosis of ATTR-CA?

□ Completing all hematological tests for the differential diagnosis of AL (serum & urine IF, sFLC); ideally before, and if not possible, simultaneously or after scintigraphy.

□ Performing CMR ideally before, and if not possible, simultaneously or after scintigraphy.

□ 99mTc-PYP imaging

□ Always performing SPECT imaging after planar imaging

□ Optimization of imaging hours based on imaging results

□ Access to SPECT-CT

□ Establishing consultant teams experienced in/focused on CA with cardiology-radiology-hematology-nuclear medicine specialists, preferably in the same institution and if not possible, in different institutions where cooperation is feasible

□ Other


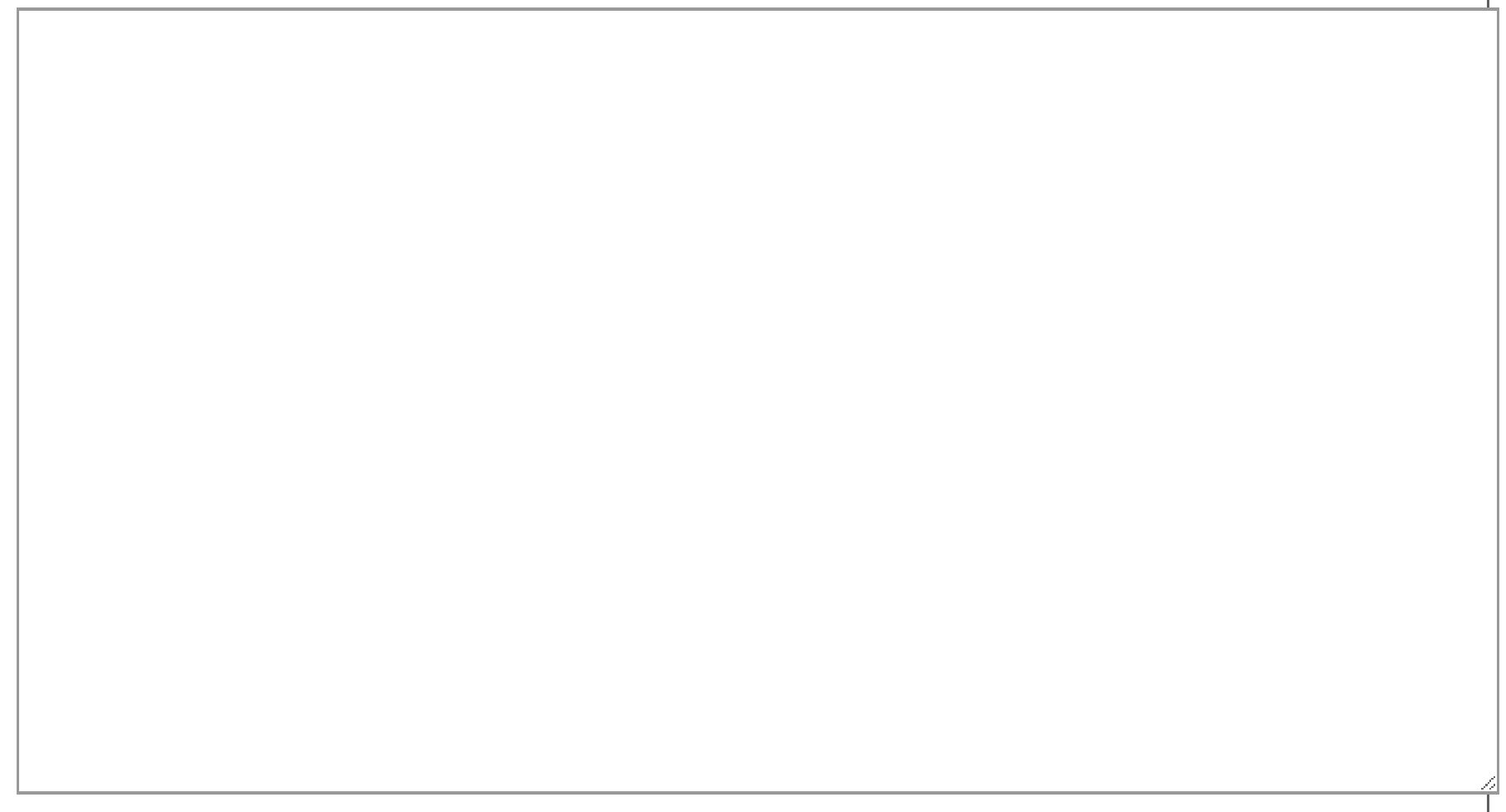

Supplement: Supplementary file 1 [file Datasheet1.docx]
